# A novel tumour enhancer function of Insulin-like growth factor II mRNA-binding protein 3 in colorectal cancer

**DOI:** 10.1038/s41419-023-05772-6

**Published:** 2023-04-06

**Authors:** Davide Di Fusco, Antonio Di Grazia, Giulia Di Maggio, Maria Teresa Segreto, Andrea Iannucci, Claudia Maresca, Alessandro De Stefano, Giuseppe Sica, Carmine Stolfi, Giovanni Monteleone, Ivan Monteleone

**Affiliations:** 1 Department of Systems Medicine, University of ‘Tor Vergata’, Rome, Italy; 2 Department of Biomedicine and Prevention, University of ‘Tor Vergata’, Rome, Italy; 3 Department of Surgery, University of ‘Tor Vergata’, Rome, Italy

**Keywords:** Colon cancer, Colon cancer

## Abstract

CRC cells evolve a variety of strategies to limit or circumvent apoptosis cell death. RNA binding proteins (RBPs) regulate many of the molecular mechanisms that underlie the development of cancer. The insulin-like growth factor II mRNA-binding proteins (IMP) family are oncofoetal RBPs, consisting of IMP1, IMP2 and IMP3, which have an important role in RNA metabolism. IMP3 is highly expressed in colorectal cancer (CRC) tissue, where its expression often correlates with poor prognosis. However, the role of IMP3 in CRC is not fully understood. IMP3 expression was analysed using a public database and by Western blotting and immunohistochemistry in human colon samples derived from patients with sporadic CRC and healthy subjects. To address whether IMP3 controls cancer cell survival, we analysed cell death pathways in in vitro and in vivo experiments after IMP3 downregulation by siRNA or an antisense oligonucleotide. IMP3 was highly expressed in CRC samples compared to normal control tissues. The knockdown of IMP3 enhanced a caspase-independent cell death in CRC cell lines. Furthermore, the treatment of CRC cells with IMP3 siRNA did not alter the expression of GSDMD, GPX-4 and the activated form of RIP3, three key molecules that govern pyroptosis, ferroptosis and necroptosis, respectively. Abrogation of IMP3 in CRC significantly reduced Bcl-2 and Bcl-xL mRNA and was associated with an altered mitochondrial membrane potential that allowed the nuclear migration of the apoptosis-inducing factor (AIF). Moreover, specific immunoprecipitation experiments on CRC human cell lines indicated that IMP3 binds Bcl-2 and Bcl-xL mRNA, suggesting that IMP3 acts as a regulator of the intrinsic apoptotic pathway through the surveillance of anti-apoptotic Bcl mRNA metabolism. Finally, we showed that IMP3 block inhibited the growth of CRC cell lines in vivo after transplantation into immunodeficient mice. Altogether, these data support a novel role for IMP3 in controlling the intrinsic caspase-independent apoptotic pathway in CRC.

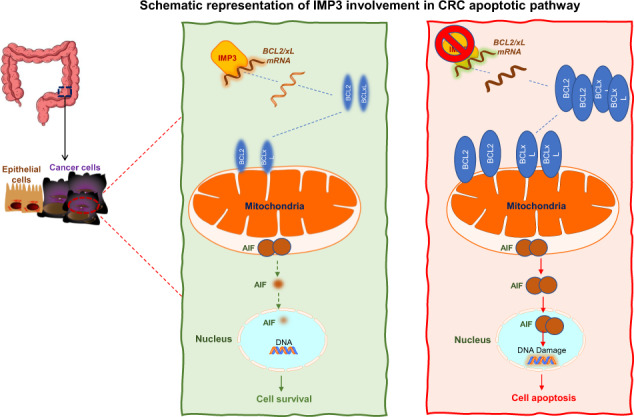

## Introduction

Colorectal cancer (CRC) is one of the most common malignant tumours worldwide and despite recent advances in CRC pathogenesis and therapy, this neoplasia is still one of the leading causes of cancer-related deaths, suggesting the necessity of effective chemopreventive/chemotherapeutic agents [1]. CRC develops in a stepwise manner from normal mucosa, to adenomatous polyps and then to carcinoma, a multistage process characterised by the accumulation of genetic changes, each conferring a selective growth advantage to tumour cells [2]. These mechanisms are determined by alterations in oncogenic and/or tumour-suppressive signalling pathways that disrupt the balance between cell proliferation and cell death that drives the progression from normal mucosa to carcinoma [2]. Oncogenic and/or tumour-suppressive expression is also determined by the effects of post-transcriptional machinery, which govern the transport/export of mRNA from the nucleus to the cytoplasm, RNA turnover, storage and translation imposed by RNA binding proteins (RBPs) [3]. Several RBPs are expressed ubiquitously and have been evolutionarily conserved to maintain their roles in basic cellular functions [4]. Any significant change or disturbance in the RBPs that regulate these essential cellular functions can lead to different diseases, including cancer [5]. The deregulation or aberrant activity of RBPs has been established to contribute to carcinogenesis [6]. Several oncogenic transcripts are protected from degradation by the binding of trans-factors on their untranslated regions (UTR). As little as 40% of the variation in protein expression can be explained by mRNA levels, indicating the importance of translation this post-transcriptional mechanism [7]. Dysregulated RBPs influence the expression and function of pro-tumorigenic and tumour suppressor proteins, among others [8]. Several studies have provided evidence that RBPs are abnormally expressed in cancer relative to adjacent normal tissues and their expression correlates with patients’ prognosis [9]. Recent studies have revealed the key contribution of several RBPs in the control of intestinal epithelial cell homeostasis [10]. A series of evidences have highlighted that tumour cells, including CRC cells, are able to highjack a series of post-transcriptional mechanisms in response to intrinsic and extracellular signals, leading to cell adaptation to the local microenvironment [8]. Several RBPs, including LIN28, MSI, CELF1, HUR and FMRP, constitute a new set of regulatory proteins that play an important role in intestinal homeostasis, adaptation to injury and participation in malignant transformation [11–15]. Moreover, the over-expression of RBPs correlates with an invasive tumour phenotype, worse survival and increased tumour recurrence in CRC [8].

Insulin-like growth factor II mRNA-binding protein 3 (IMP3), an RBP involved in multiple steps of mRNA metabolism, is gaining pivotal importance in controlling the development and growth of different types of human cancer [16]. IMP3 is frequently expressed in many different tumour types and has typically been associated with aggressive tumour features [16]. Moreover, in a recent paper, Tschirdewahn and colleagues found that serum IMP3 levels associated with poor survival in renal cell carcinoma (RCC) patients, thus suggesting its role as a biomarker to improve the risk-stratification of RCC patients and to optimise therapeutic and follow-up decisions [17]. Also in CRC, IMP3 expression is associated with adverse clinical outcomes and as prognostic biomarker [18]. A recent systematic review suggested that high IMP3 expression was associated with worse overall survival in patients with solid tumours and this negative prognostic effect was also specifically observed in CRC [19]. Furthermore, a recent article indicated a shorter overall survival between patients with rectal cancer and high IMP3 protein levels respect to the same patients without or with low levels of IMP3 expression [20], proposing IMP3 evaluation by immunohistochemistry as an independent prognostic factor of the clinical stage of rectal cancer [20]. However, despite several epidemiological studies suggesting negative prognostic effects of IMP3 expression in CRC, little is known about the molecular mechanisms that support its role. Two recent studies have suggested that IMP3 regulates the progression of colorectal cancer by activation of MEKK1/ERK pathway and subsequent interaction with ELAVL1 [21, 22]. Thus, a better understanding of the contribution of IMP3 in CRC could help clinicians to personalize the therapeutic strategies in order to contrast with neoplastic growth and disease progression. In this study, we assessed the role of IMP3 in human sporadic CRC cells. We observed that IMP3 down-regulation shows a protective effect toward cancer progression and identified the underlying molecular mechanism based on the control of *Bcl-2* and *Bcl-xL* transcripts, two key mediators of the intrinsic apoptotic pathway.

## Results

### IMP3 is up-regulated in human CRC tissues and correlates with poor prognosis in CRC patients

**Fig. 1:**
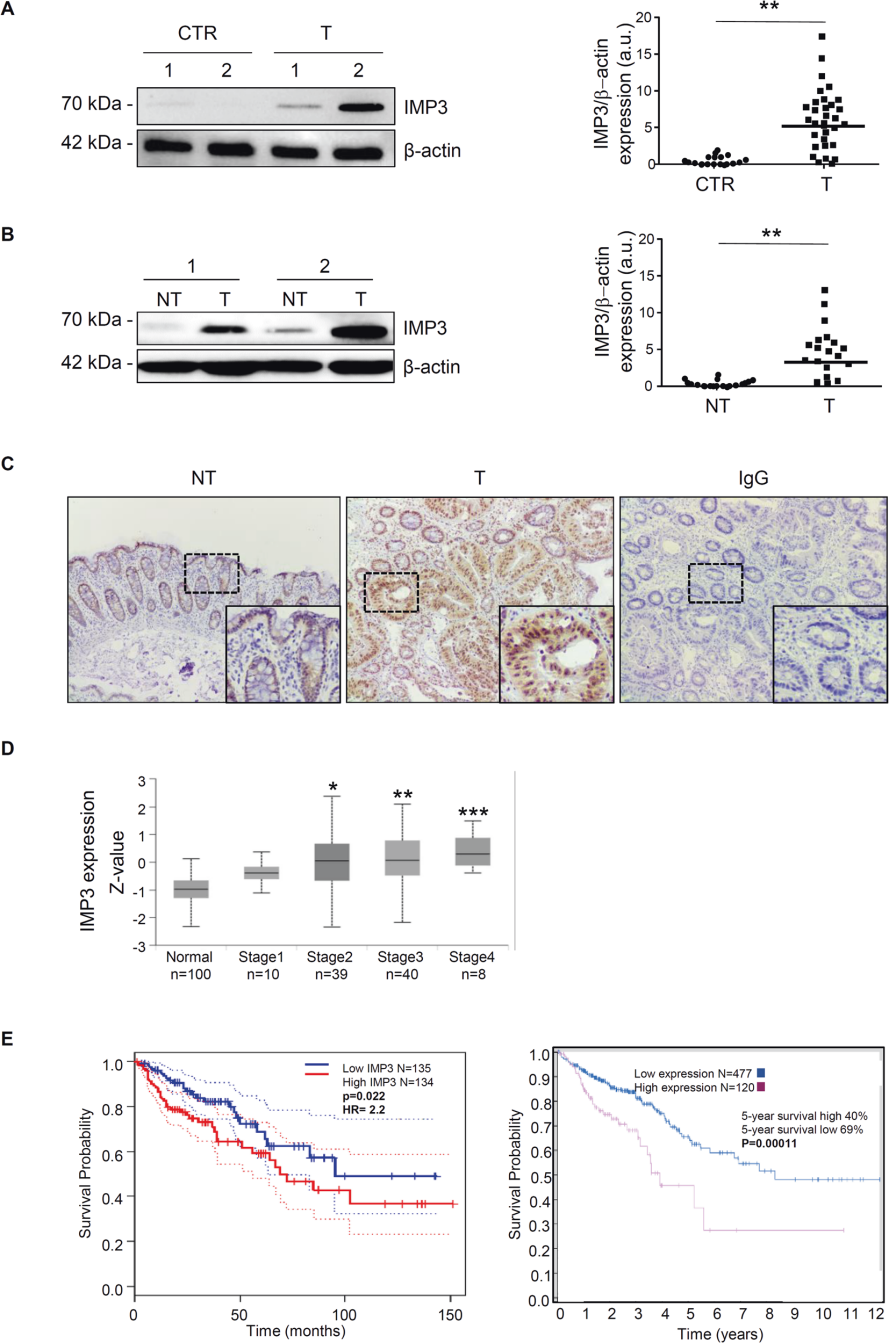
IMP3 is overexpressed in human sporadic CRC. **A** Left, IMP3 levels in representative images of Western blot from colonic samples derived from two control subjects (CTR) and two patients with CRC (T). β-actin was used as a loading control. Right inset shows quantitative analysis of IMP3/β-actin protein ratio as measured by densitometry scanning of all Western blots in which 32 CRC patients and 18 controls were included. Each point in the graph indicates the value of IMP3/β-actin in each patient (values are expressed in arbitrary units (a.u.); CTR versus T, **p ≤ 0.01). **B** Total proteins extracted from both tumoral (T) and non-tumoral (NT) areas of two patients with sporadic CRC were evaluated for IMP3 expression by Western blotting. β-actin was used as loading control. Right inset shows quantitative analysis of IMP3/β-actin protein ratio as measured by densitometry scanning of all Western blots in which 19 CRC patients were included. Each point in the graph indicates the value of IMP3/β-actin in each patient (values are expressed in arbitrary units (a.u.); NT versus T, **p ≤ 0.02). **C** IMP3 immunostaining in specimens taken from tumoral (T) and non-tumoral (NT) areas of one patient with sporadic CRC. The figure is representative of four separate experiments in which sections of four CRC patients were analyzed. Staining with isotype control IgG is also shown. **D** Box plots evaluating IMP3 expression among different groups of patients based on clinical parameters using the UALCAN database. Analysis is shown for cancer stage. *p ≤ 0.05, **p ≤ 0.01, ***p ≤ 0.001. **E** Survival curve evaluating the prognostic value of IMP3 expression. Survival curves using the Kaplan-Meier plotter are shown for overall survival using GEPIA (right panel) and Human protein atlas database (left panel).

To address the question of whether IMP3 is involved in the survival of patients with CRC, different publicly available datasets were screened for IMP3 expression levels. In the Tumour Immune Estimation Resource (TIMER; http://timer.cistrome.org/) [23], a high *IMP3* mRNA expression was found in different solid human cancers, including CRC, compared with the corresponding normal tissues (Supplementary Fig. 1A), as well as in the Gene Expression Profiling Interactive Analysis (GEPIA; http://gepia.cancer-pku.cn/index.html) [24] and UALCAN databases (http://ualcan.path.uab.edu/) [25] (Supplementary Fig. 1B, C). Firstly, we have examined IMP3 protein expression in tumour and control mucosa of CRC patients of our cohort by Western blotting and immunohistochemistry. Consistently, IMP3 was found to be highly expressed in approximately 75% of colon cancer samples (T) compared to healthy subjects (CTR) (Fig. 1A). IMP3 expression was also significantly increased in paired tissue samples derived from the tumoral area (T) respect with macroscopically unaffected, adjacent colonic mucosa (NT) of 19 patients CRC patients (Fig. 1B). These findings were confirmed by immunohistochemistry, revealing an increase of the number of IMP3-positive cells in lamina propria and epithelium compartments of CRC compared with NT tissues (Fig. 1C). Next, to investigate IMP3 expression among groups of patients according to different CRC clinical parameters, the bioinformatic analysis by UALCAN online tool was performed. Regarding CRC tumour stage, a statistically significant increase of IMP3 expression was detected in patients belong to 1, 2, 3 and 4 stage of the disease (Fig. 1D). Moreover, by GEPIA database, we examined the prognostic value of IMP3 expression in CRC patients. CRC patients with substantial IMP3 gene expression exhibited a poor overall survival (OS) according to the Kaplan-Meier plotter database for survival analysis (Fig. 1E), as well as reported by Human Protein Atlas database (https://www.proteinatlas.org/; Fig. 1E).

These findings clearly illustrate that IMP3 expression is upregulated in CRC and associates with a poor prognosis in CRC patients.

### IMP3 affects survival in human CRC cells

**Fig. 2:**
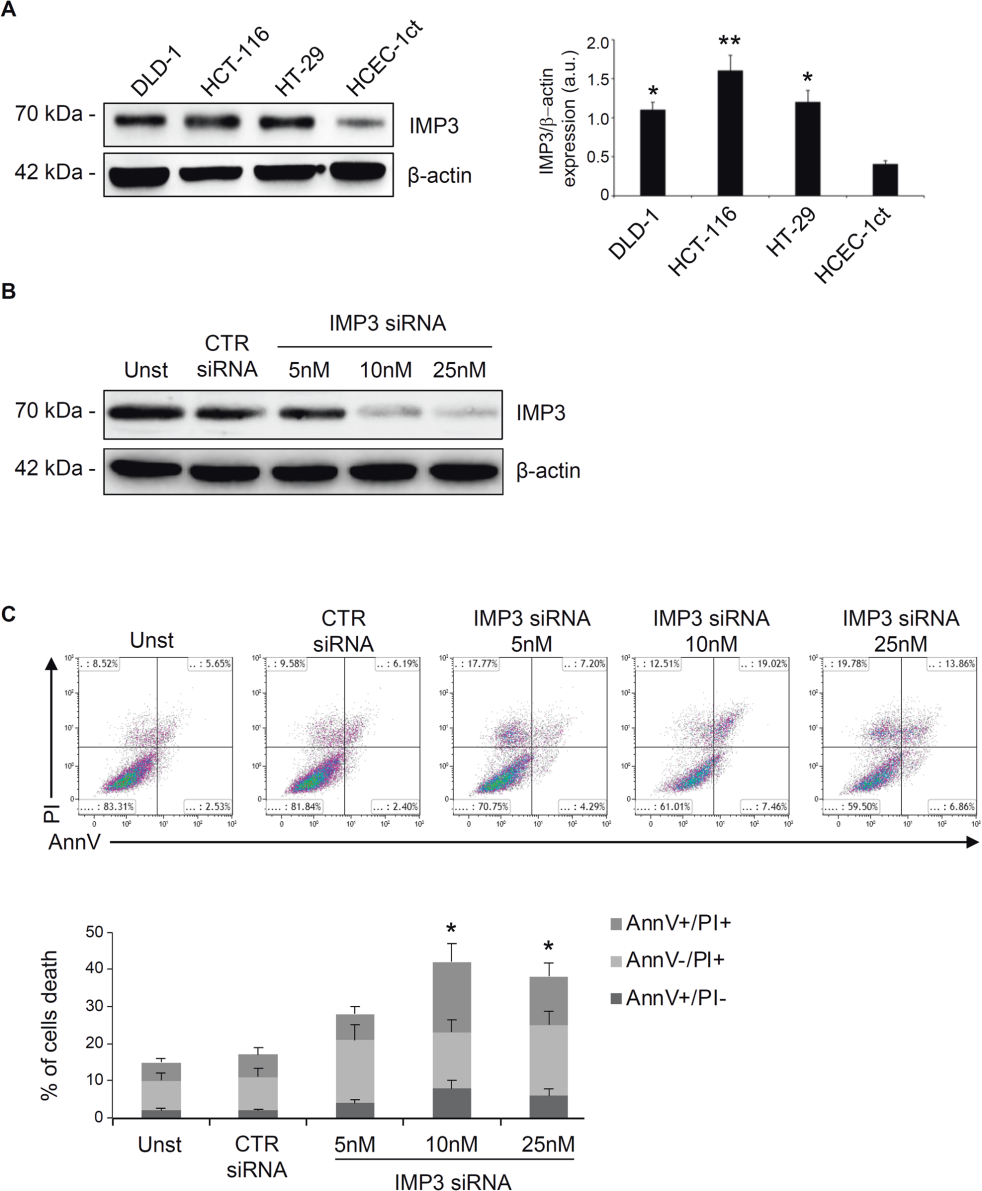
Knockdown of IMP3 triggers cell death in CRC cell lines. **A** Left, IMP3 levels in representative images of Western blot from DLD-1, HCT-116 and HT-29 CRC cell lines and the healthy colon epithelial cell line HCEC-1ct. β-actin was used as loading control. Right, quantitative analysis of IMP3/β-actin protein ratio as measured by densitometry scanning of Western blots (values are expressed in arbitrary units a.u.; *p = 0.04, **p = 0.03; mean ± SD, *n* = 3). **B** Representative Western blot showing IMP3 expression in HCT-116 cells untreated (Unst) or transfected with control or IMP3 siRNA. **C** Upper panel, representative dot plot of Annexin V (AnnV) and propidium iodide (PI)-positive HCT-116 cells treated as indicated above for 48 h. Lower panel, quantification of the percentage of AnnV and/or PI-positive HCT-116 cells (mean ± SD; AnnV+PI + unst and CTR siRNA-treated cells versus IMP3 siRNA 10/25nM-transfected cells, *p ≤ 0.05, mean ± SD, *n* = 3).

Since IMP3 has emerged as a critical regulator of multiple important targets and pathways involved in cell survival [26], we examined whether IMP3 could act as a key regulator of cancer cell survival. Therefore, we have investigated CRC cell death in vitro in presence or absence of IMP3. In initial experiments, IMP3 protein expression was evaluated in three different CRC cell lines (DLD-1, HCT-116 and HT-29) and in non-cancer colonic epithelial cell line HCEC-1ct by Western blotting. There was a more pronounced IMP3 expression in all CRC cell line compared with HCEC-1ct, with no significant difference in terms of IMP3 expression between DLD-1, HCT-116 and HT-29 cells, even if a slight increase was detected in HCT-116 (Fig. 2A). Therefore, we have chosen these cells in the subsequent experiments.

**Fig. 3:**
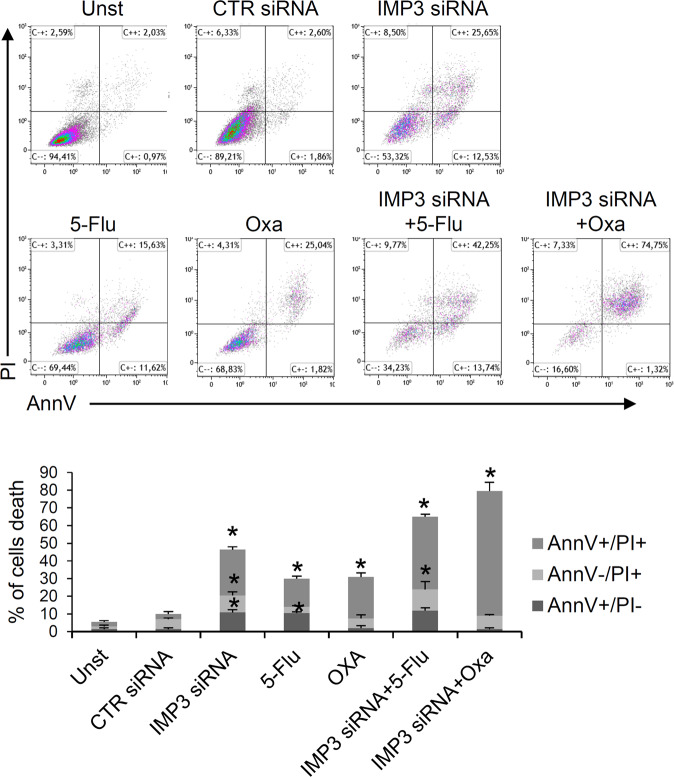
IMP3 knockdown enhances the toxicity of chemotherapeutic drugs. Representative dot-plots (upper panels) and quatification percentage (lower panel) showing the AnnexinV- and/or propridiun iodide (PI)-positive HCT-116 cells pre-incubated with control or IMP3 siRNA (both final concentration 25 nM) for 12 h and then stimulated or not with 5-fluorouracil (5-flu, 10uM final concentration) or Oxaliplatin (oxa, 10uM final concentration) for 36 h. Data indicate mean ± SEM of 2 separate experiments (CTR siRNA-treated cells versus all, *p ≤ 0.01).

Treatment of HCT-116 cells with 2 different selective siRNA of IMP3 significantly reduced IMP3 protein expression (Fig. 2B and supplementary Fig. 2A). HCT-116 cells treated with the IMP3 siRNA showed an increased susceptibility to spontaneous cell death; in particular, most CRC cells appear to be AnnV+/PI+, the typical flow cytometric label of programmed cell death (Fig. 2C and supplementary Fig. 2A). Similar results were also obtain in DLD-1 and HT-29 cell line and did not occur in normal cells HCEC-1ct suggesting a cancer cell type-specific phenomenon (supplemental Fig. 2B, C). Furthermore, we explored the possibility that IMP3 levels confers chemoresistance advantage to CRC cells. As shown in Fig. 3, treatment of HCT-116 cells with IMP3 siRNA increased the sensibility of CRC cells to 5-fluoruoracil and oxaliplatin, suggesting that IMP3 knockdown could potentially be deployed as an anti-cancer drug in the combinatorial approaches with chemotherapeutic drugs.

**Fig. 4:**
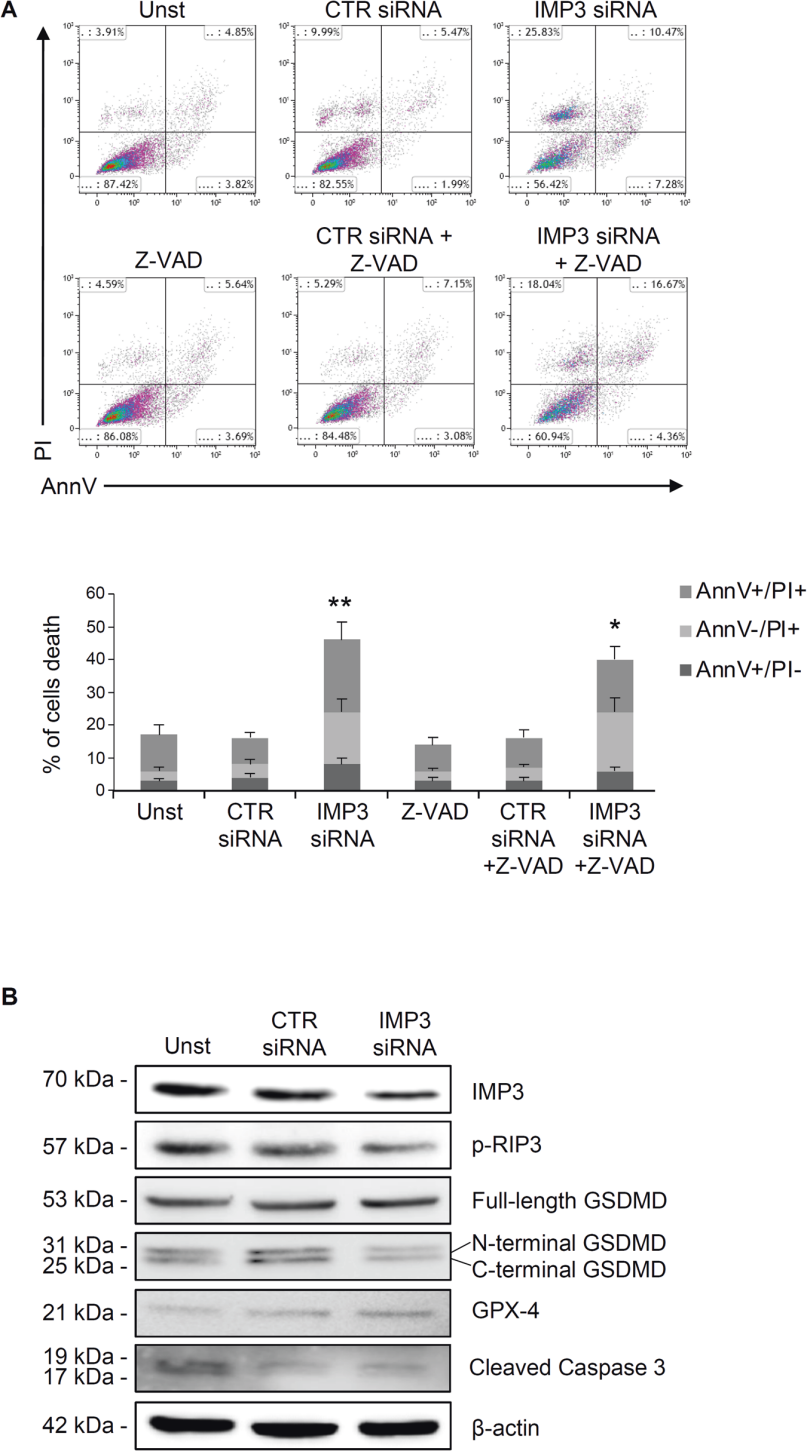
IMP3-triggered CRC cell death is independent by caspases activation and does not affect necroptosis, pyroptosis or ferroptosis pathways. **A** Upper panel, representative dot plot showing Annexin V (AnnV) and propidium iodide (PI)-positive HCT-116 cells pre-incubated with pan-caspase inhibitor (Z-VAD) and left untreated (Unst) or transfected with control or IMP3 siRNA for 48 h. Lower panel shows the percentage of AnnV and/or PI-positive HCT-116 treated as above (mean ± SEM; AnnV+PI + unst and CTR siRNA-treated cells versus IMP3 siRNA-transfected cells, **p ≤ 0.01; Z-VAD-cells and Z-VAD CTR siRNA-treated cells versus Z-VAD IMP3 siRNA-treated cells, *p ≤ 0.05, *n* = 4). **B** Representative Western blot of activated form of RIP3 (p-RIP), Gasdermin D (GSDMD), Glutathione peroxidase 4 (GPX4) and cleaved capsase 3 in HCT-116 cells left untreated (Unst) or transfected with control or IMP3 siRNA for 48 h. β-actin was used as loading control (*n* = 3).

To dissect the molecular mechanism implicated in IMP3 siRNA-induced cell death, we investigated the involvement of the main mechanisms related to programmed cell death. Specifically, pre-treatment of HCT-116 cells with a pan-caspase inhibitor (Z-VAD) did not alter IMP3 siRNA-induced cell death (Fig. 4A). Moreover, the treatment of CRC cells with IMP3 siRNA did not alter the expression of activated caspase 3, 8 and 9 (Fig. 4B and supplementary Fig. 2D). In addition, IMP3 silencing in these cells did not modified the expression of activated form of RIP3 (p-RIP3), GSDMD and GPX-4, three key molecules which orchestrate necroptosis, pyroptosis and ferroptosis pathways, respectively (Fig. 4B). Furthermore, pre-treatment of HCT-116 cells with deferoxamine, a ferroptosis inhibitor or disulfiram, a pyroptosis inhibitor, necrostatin-1, a necroptosis inhibitor, or a pan-caspase inhibitor (Z-VAD) alone or combined with each other did not alter IMP3 siRNA-induced cell death (supplemental Fig. 3). Altogether, these findings suggest that IMP3 influences CRC cell survival without affecting classical caspase-dependent or alternative programmed cell death mechanisms.

### IMP3 regulates Bcl-2/Bcl-xL expression

**Fig. 5:**
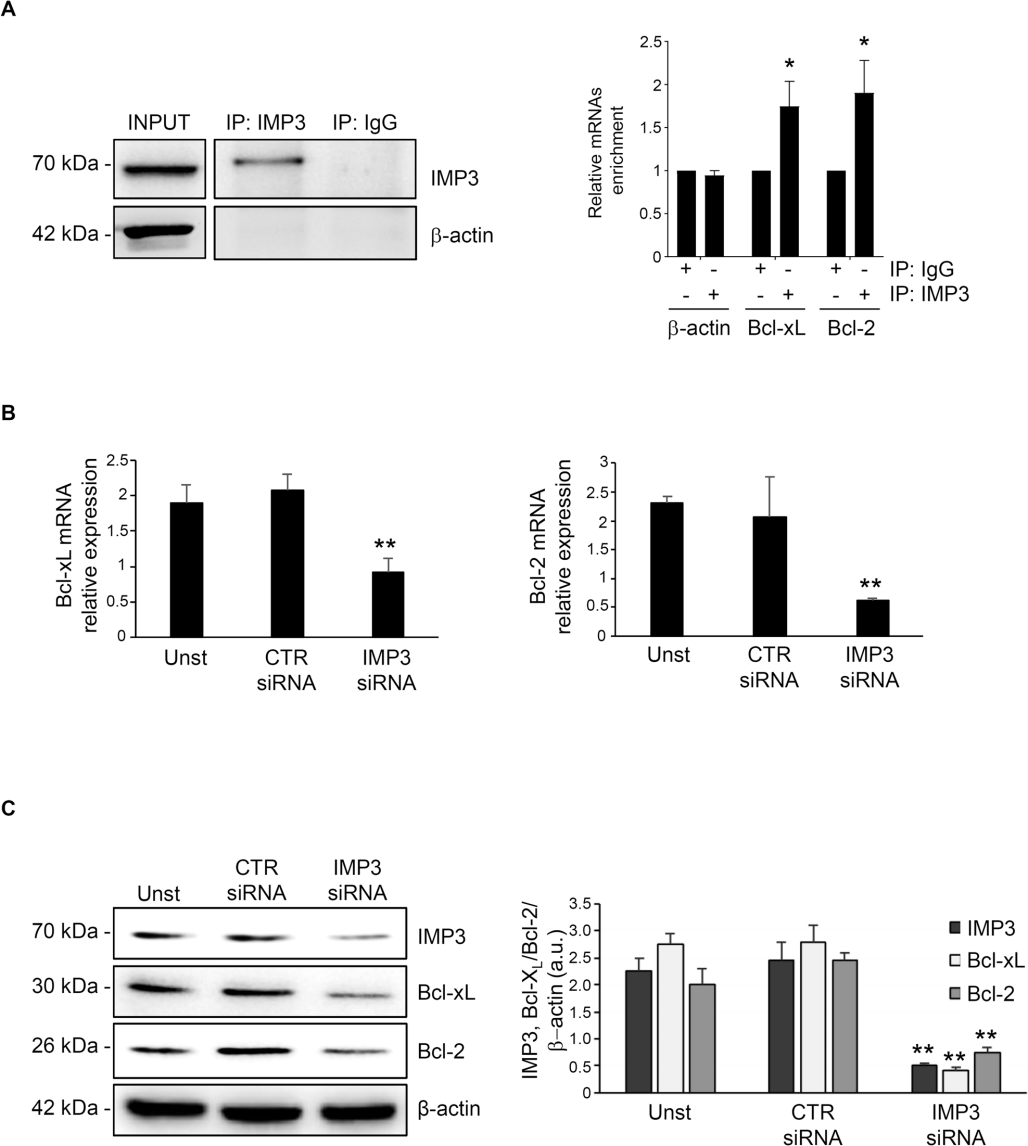
*Bcl-2* and *Bcl-xL* mRNA are part of the IMP3 complex. **A** Left, representative Western blot of IMP3 immunoprecipitation from HCT-116 cells, β-actin is used as a negative control. Input (1:10) of the total protein extracts, IMP3 immunoprecipitation (IP: IMP3), and mock immunoprecipitation (IP: IgG). Right, relative quantification of IMP3-mRNAs enrichment by RT-qPCR in IMP3 immunoprecipitation/total protein extracted from HCT-116 cells*. β-actin* mRNAs were used as control (mean ± SEM; IP: IMP3 versus IP: IgG *P ≤ 0.05; *n* = 3). **B** *Bcl-xL* and *Bcl-2* mRNA levels detected by Real time PCR in HCT-116 cells left untreated (Unst) or transfected with CTR or IMP3 siRNA for 30 h. Unst and CTR siRNA-treated cells versus IMP3 siRNA-transfected cells, **p ≤ 0.01; *n* = 3). **C** Representative Western blot showing IMP3, Bcl-xL, Bcl-2 and β-actin in HCT-116 cells were left untreated (Unst) or transfected with control or IMP3 siRNA for 36 h. Right, quantification of IMP3, Bcl-xL and Bcl-2 proteins in HCT-116 cells as measured by densitometry of Western blot (values are expressed in arbitrary units (a.u.), mean ± SD, *n* = 4; IMP3: Unst and CTR siRNA-treated cells versus IMP3 siRNA-transfected cells, **p ≤ 0.01; *n* = 3).

The mitochondria are known to be a central relaying station for caspase-independent death pathways in which Bcl proteins, located in the outer mitochondrial membrane, represent the main actors in controlling the mitochondria-associated apoptotic intrinsic pathway [27]. All mitochondrial activities can be blocked by the over-expression of Bcl anti-apoptotic proteins in experimental models of caspase-independent apoptosis [28]. A database for exploring Post-transcriptional Regulation, based on high-throughput sequencing data from human tissue, provides the largest binding site collection of RPBs and their possible mRNA targets (POSTAR3; http://111.198.139.65/index.html) [29]. By a carefully screening on POSTAR database, coding transcripts of *Bcl-2* and *Bcl-xL* were resulted as possible targets of IMP3. Therefore, we explored the possibility that IMP3 could bind *Bcl-2* and *Bcl-xL* mRNAs, known to be highly expressed in CRC tissue and with crucial role in cancer progression and therapy resistance [30]. To confirm this, IMP3 was immunoprecipitated from total lysates extracted from HCT-116 and DLD-1 cells and the association of these selected mRNAs tested by RT-qPCR. A significant enrichment in the IMP3 complex from HCT-116 and DLD-1 cell lines was detected for *Bcl-2* and *Bcl-xL* mRNA but not for β-*actin* mRNA that was used as negative control (Fig. 5A and supplementary Fig. 4). To examine whether IMP3 is responsible to maintain reduced levels of the anti-apoptotic Bcl molecules in CRC, we explored Bcl-2 and Bcl-xL expression in HCT-116 cell line treated with or without IMP3 siRNA. Effective silencing of IMP3 in HCT-116 inhibited IMP3 expression and this reduction associated with *Bcl-2* and *Bcl-xL* downregulation at mRNA and protein levels (Fig. 5B, C). To further confirm that pro-survival function of IMP3 in CRC cells is mediated by *Bcl-2* and *Bcl-xL* expression, we explored the impact of down-regulating or over-regulating *Bcl-2* and *Bcl-xL* on cell death. HCT-116 cells treated with the Bcl-2 or Bcl-xL siRNA alone or in combination showed an increased susceptibility to spontaneous cell death (supplementary Fig. 5A). Conversely, HCT-116 overexpressing *Bcl-2* and/or *Bcl-xL* showed an increased resistance to IMP3 siRNA-induced cell death (supplementary Fig. 5B). Our findings indicate a pro-survival function of IMP3 in CRC cells through Bcl-2 and Bcl-xL mRNAs binding.

### IMP3 regulates cell death by mitochondrial intrinsic apoptotic pathway

**Fig. 6:**
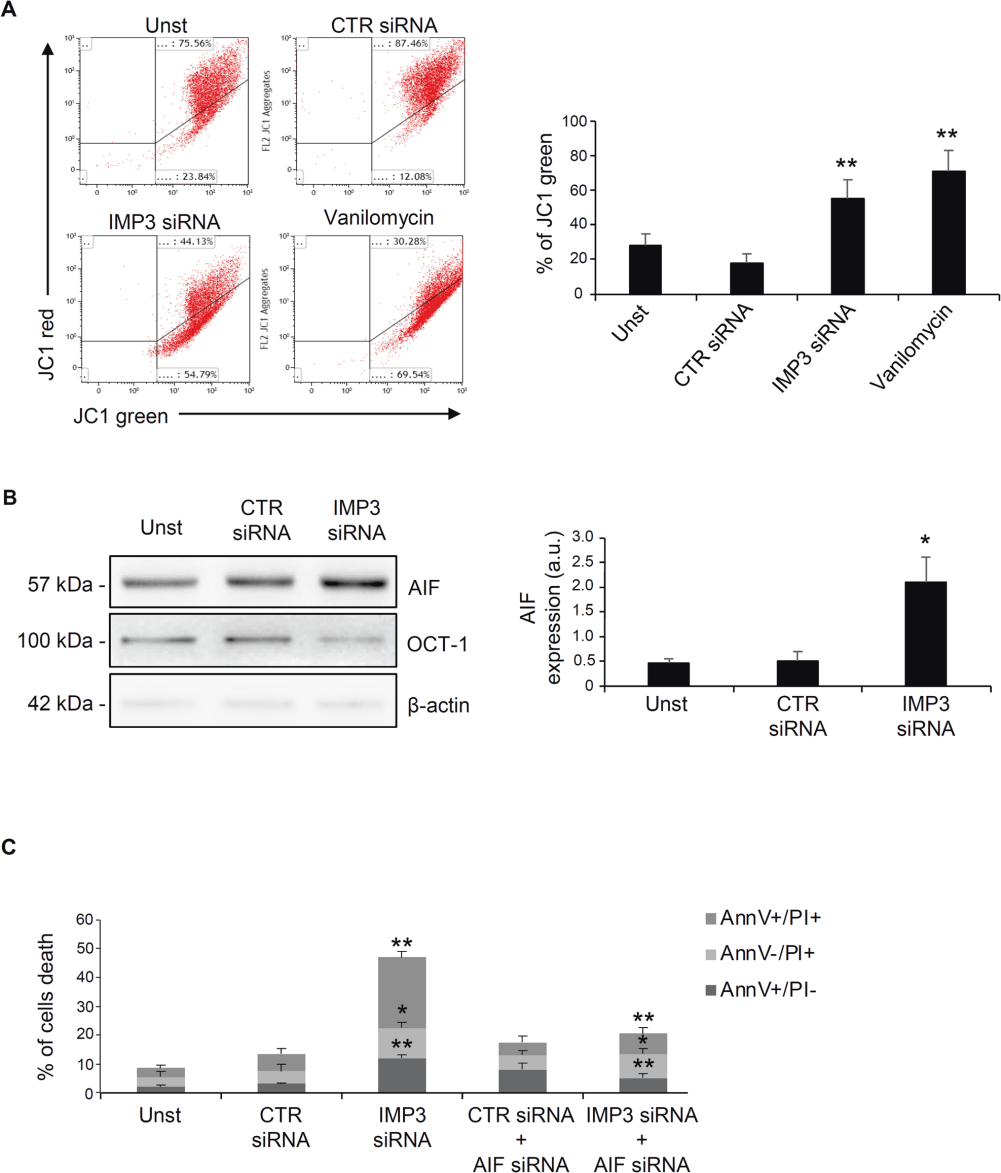
IMP3 knockdown affects mitochondrial membrane potential and AIF nuclear accumulation. (**A**) HCT-116 cells were left untreated (Unst) or transfected with CTR or IMP3 siRNA. Vanilomycin was used as positive control. HCT-116 were stained with JC-1 and analyzed by flow cytometry. Mitochondrial membrane potential loss was evaluated as a decrease in JC-1 red fluorescence and an increase in JC-1 green fluorescence. Representative dot plot (Unst and CTR siRNA-treated cells versus IMP3 siRNA-transfected cells and Unst cells versus vanilomycin-treated cells, mean ± SD, **P ≤ 0.01; *n* = 3). **B** Representative Western blot (left panel) and quantitative analysis (right panel) for AIF, OCT-1 and β-actin in nuclear protein extracted from HCT-116 cells untreated (Unst) or transfected with CTR or IMP3 siRNA for 48 h. (Unst and CTR siRNA-treated cells versus IMP3 siRNA-transfected cells, mean ± SD, **p ≤ 0.01; *n* = 3). **C** Percentage of AnnV and/or PI-positive HCT-116 cells untreated (Unst) or transfected with CTR or IMP3 siRNA in presence/absence of AIF siRNA for 48 h (CTR siRNA-treated cells versus IMP3 siRNA-transfected cells, *p = 0.04, **p ≤ 0.01;and IMP3 siRNA-transfected versus IMP3 siRNA + AIF siRNA transfected cells *p = 0.04, **p ≤ 0.01; mean ± SD, *n* = 3).

In intrinsic apoptosis pathway, Bcl-2 and Bcl-xL play a key role in determining the decision of cells to undergo apoptosis by affecting the mitochondria-associated membrane potential [28]. Therefore, we explored whether IMP3 siRNA-induced cell death was secondary to mitochondrial membrane permeabilization (MMP) after *Bcl-2* and *Bcl-xL* down-regulation. In our experiment valinomycin was used as a positive control to dissipate MMP. In HCT-116 cells, IMP3 silencing triggered a rapid loss of MMP, similar to valinomycin-treated cells (Fig. 6A). To further determine the role of mitochondria-mediated death processes in our system we examined the activation of Bax in the mitochondria, using a conformation-specific antibody that specifically detects the active form of Bax. IMP3-silenced cells showed an increased percentage of activated Bax positive cells respect to control siRNA-treated cells (supplementary Fig. 6A). Changes to the outer MMP cause the outflow of several molecules involved in apoptosis caspase dependent. One of the soluble factors released from perturbed mitochondria, which is capable of triggering caspase-independent apoptosis, is an inter-mitochondrial membrane protein called AIF. Upon specific apoptotic stimuli, AIF undergoes to proteolytic cleavage and translocates to the nucleus, triggering chromatin condensation and a large-scale DNA degradation in a caspase-independent manner [31]. For this purpose, we have assessed whether IMP3 siRNA-induced apoptosis was a consequence of AIF nuclear migration in nuclear protein lysates isolated from CRC cells. As expected, IMP3-silenced cells showed an increased AIF nuclear expression respect with control siRNA-treated cells (Fig. 6B).

To confirm whether IMP3-induced cell death was specifically dependent by AIF activation, HCT-116 cells were incubated with or without a selective AIF siRNA. SiRNA of AIF significantly reduced AIF protein expression (supplementary Fig. 6B) and CRC cell death induced by *IMP-3* silencing was completely reversed in the presence of AIF siRNA (Fig. 6C).

These data indicate that IMP3 siRNA-induced cell death in CRC cells is due to activation of the intrinsic apoptotic pathway and the subsequent AIF nuclear migration.

### IMP3 knockdown reduces the in vivo growth of HCT-116-derived xenografts

**Fig. 7:**
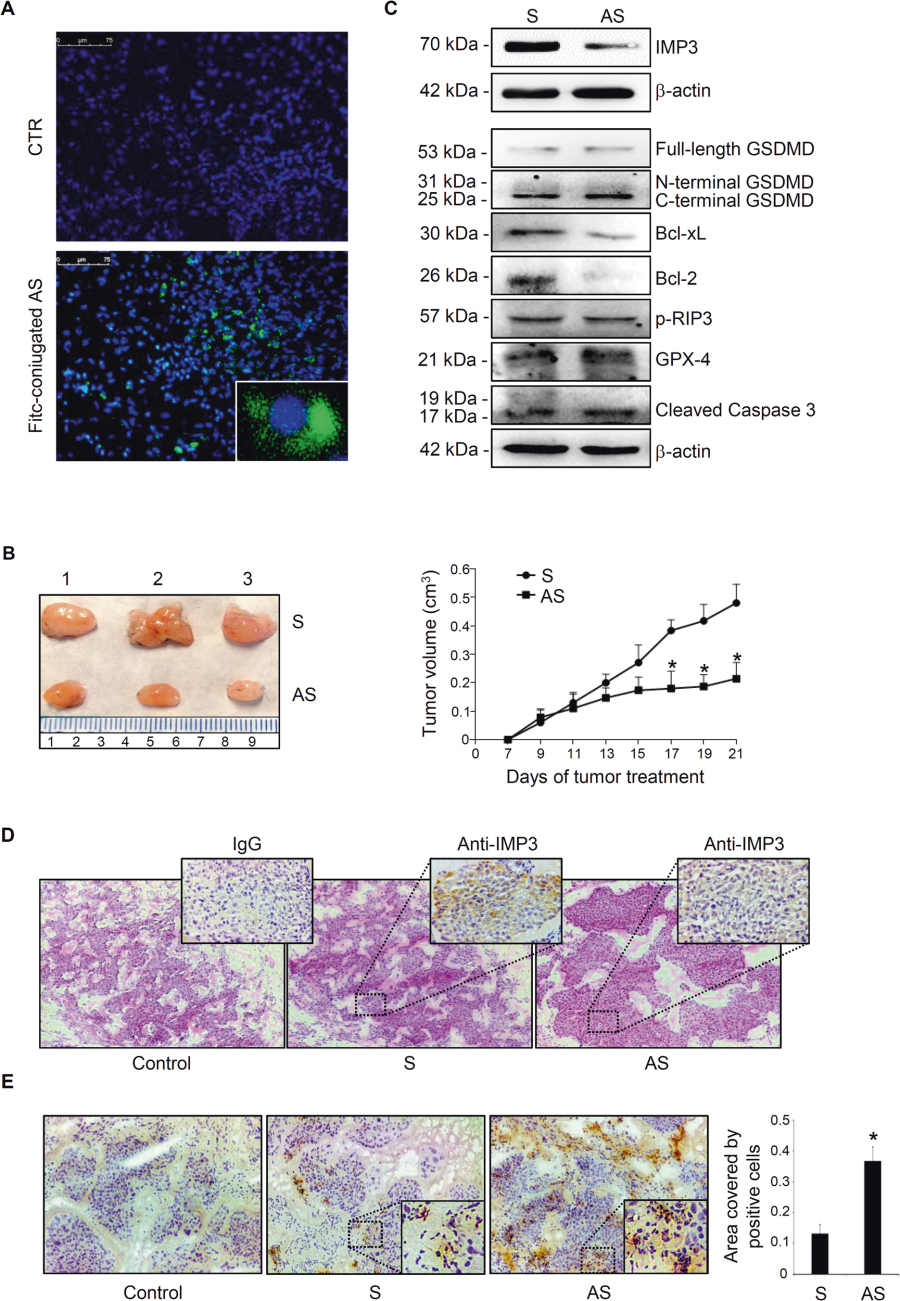
Effect of IMP3 knockdown on the in vivo formation of HCT-116-derived xenografts. **A** Representative images showing the uptake of fluorescein isothiocyanate (FITC)-conjugated IMP3 antisense (AS) oligonucleotide in HCT-116-derived xenografts induced in *Rag1*^-/-^ mice receiving a single intraperitoneal injection of either control (PBS) or AS (100 μg). Positive cells were evaluated by immunofluorescence. **B** Representative photographic images (left panel) and relative histograms (right panel) showing the volume of HCT-116-derived tumors taken from mice treated with Sense (S) or Antisense (AS) oligonucleotide. HCT-116 were injected subcutaneously in *Rag1*^-/-^ mice and after one week, intraperitoneal injected with S (control), or IMP3 AS (both at concentration of 100 μg/mouse/day). Data of tumor volume (cm^3^) were reordered every two days starting from day 7. Mice were sacrificed at day 21. Data indicate the mean ± S.D. in which 5 mice per group were included. (*p ≤ 0.04; S-treated versus AS-treated mice). **C** Representative Western blot of IMP3, Bcl-xL, Bcl-2, p-RIP3, GSDMD, GPX-4 and cleaved caspase 3 expression in HCT-116-derived xenografts induced in Rag1^-/-^ mice and treated as indicated above. β-actin was used as a loading control. **D** Representative pictures of H&E and immunohistochemistry for IMP3 in tumours excised from mice treated ad above. Control isotype (IgG) is also shown. **E** Tunel-stained sections of HCT-116-derived xenografts taken from mice treated with S or AS. Control isotype is also shown. Right inset indicates the quantification of positive cells in tumour area. Values indicate mean ± S.D. (*p ≤ 0.05; S-treated versus AS-treated mice).

Finally, to confirm the anti-neoplastic effect of IMP3 silencing, we tested the ability of IMP3 knockdown to inhibit tumour growth in vivo. Downregulation of IMP3 expression anti-sense oligonucleotide (AS)-induced was previously tested in vitro experiments (data not shown). HCT-116 cells were subcutaneously inoculated into *Rag1*^-/-^ mice and after one week, the animals received a single intraperitoneal injection of either PBS (CTR) or fluorescein isothiocyanate (FITC)-conjugated IMP3 AS. Twenty-four hours later, mice were killed, tumours excised and the distribution of labelled IMP3 AS assessed by immunofluorescence. As showed in Fig. 7A IMP3 AS was efficiently taken up by growing tumour cells into the mice flank. Next, *Rag1*^-/-^ mice have subcutaneously received HCT-116-derived xenografts and were treated intraperitoneally with either IMP3 S or AS (both at 100 μg/mouse daily) starting 7 days after the HCT-116 injection until euthanisation (day 21). No weight loss was observed in either treated group and all animals survived until the end of the experimental procedure. At day 21 HCT-116-derived tumours were visible in all mice. The in vivo inhibition of IMP3 with a specific AS reduced tumour volume and paralleled by decreased IMP3 protein expression in the tumour mass (Fig. 7B–D). In addition, consistently with our results obtained in vitro, IMP3 silencing reduced Bcl-2 and Bcl-xL expression but did not modified the expression of activated form of RIP3, GSDMD, GPX-4 and caspase 3. Moreover, to investigate whether the decreased HCT-116 derived tumor volume observed in IMP3 AS treated mice was due to an increase in cell death, we performed a terminal deoxynucleotidyl transferase dUTP nick end labeling (TUNEL). IMP3-knockdowned cells exhibited increased number of cell death (tunel positive cells), thus recapitulating the major finding seen in vitro experiments (Fig. 7E).

## Discussion

The development of CRC is a multifactorial process with environmental, epigenetic and genetic factors being implicated [32]. Compelling evidence indicates that CRC cells are marked by the enhanced activation of various intracellular signals, which ultimately promote the expression of molecules involved in cell survival/anti-apoptosis and cell cycle progression. In recent years, a better understanding of these deregulated pathways led to the development of therapeutic strategies, which have been employed with some success in CRC patients [33]. Among the factors that ensure the correct development of intestinal cells are the family of RBPs. Increasing evidence indicates that the response and adaptation of intestinal epithelium to various types of injuries are mediated by RBPs [11]. A single RBP can bind to hundreds, if not thousands, of mRNA targets and a combination of several RBP interactions contribute to cellular identity in healthy cells, as well as in pathological conditions such as cancer [34].

Here, we show that IMP3 expression is significantly increased in human CRC compared with normal colon cells. Bioinformatic analysis of public databases revealed a high IMP3 expression level in CRC tissues, further confirming and extending our observation. Subsequently, the clinical prognostic significance of IMP3 in CRC was investigated using public cancer databases. The high expression of IMP3 was significantly correlated with histological grade and metastasis in CRC patients. Furthermore, Kaplan-Meier survival analyses indicated that CRC patients with high IMP3 expression exhibited a markedly worse survival rate than those with low expression of IMP3. These results substantiated that IMP3 may be an independent prognostic biomarker in CRC and could facilitate the development of targeted precision oncology therapies. These data are in accordance with previous findings indicating that IMP3 levels are predictive of poor survival in multiple solid tumours [18–20].

Resistance to cell death is a crucial hallmark acquired during cancer progression. CRC cells have evolved a variety of strategies to limit or evade programmed cell death and tumour cells may block the apoptosis process by increasing the expression of antiapoptotic regulators such as Bcl-2 and Bcl-xL, by down-regulating pro-apoptotic factors or by short-circuiting the extrinsic ligand-induced death pathway [2]. The identification of deregulated pathways affecting cell death has led to the development of therapeutic strategies that have been used in CRC patients [33]. Several RBPs that are up-regulated in cancer tissue can modulate the expression of genes implicated in cell survival [11, 34], which prompted us to hypothesise that IMP3 could control programmed cell death also in CRC cells. IMP3 knockdown affects survival in human CRC cell lines both in vitro and in vivo; however, the inhibition of caspases and unmodified expression levels of three key molecules involved in ferroptosis, necroptosis and pyroptosis pathways of programmed cell death, were not influenced. These findings suggest that the anti-survival effect of IMP3 siRNA is not due to caspase activation or ferroptotic, pyroptotic or necroptotic mechanisms. Several lines of evidence are drawing attention to the emergent role of cell death pathways where caspases are not involved; recent advances have revealed that mitochondria can play an central role via the release of proapoptotic factors that induce caspase-independent programmed cell death [35]. The alteration of MMP, which is controlled by the Bcl-2 family of proteins including Bcl-2 and Bcl-xL, is a critical event in apoptosis. Here, we demonstrated that CRC cells incubated with IMP3 siRNA regain the normal activity of the machinery that drives caspase-independent programmed cell death. Specific immunoprecipitation experiments show that IMP3 binds *Bcl-2* and *Bcl-xL* mRNA, suggesting that IMP3 acts as a regulator of the intrinsic apoptosis pathway, ultimately affecting Bcl-2 and Bcl-xL synthesis. In this study, for the first time, we have detected the presence of *Bcl-2* and *Bcl-xL* mRNAs in the IMP3 complex, indicating that IMP3 can regulate their metabolism. Interestingly, using a webserver database (POSTAR3) for prediction of mRNA target, we found that *Bcl-2* and *Bcl-xL* mRNA represent a possible IMP3 binding site. However, this is a predictive approach and RBPs could create an intricate system for controlling gene expression at the post-transcriptional level [36]; therefore, future studies should further investigate how IMP3 could control *Bcl-2* and *Bcl-xL* splicing, stability or subcellular localisation after mRNA binding.

In classical caspase-dependent apoptosis, after MMP induction cytochrome C redistributes from the mitochondria to the cytosol and activates caspase-9, in collaboration with ATP and the cytosolic factor Apaf-1 [37]. However, several studies demonstrated that altered expression of inhibitor of apoptosis proteins (IAP) family members, family of antiapoptotic proteins that bind and inhibit caspases 3, 7, and 9, is frequent in colorectal carcinoma [38, 39]. However, the intermembrane space of the mitochondria also contains other proteins, such as AIF, which can provoke programmed cell death in a caspase-independent manner [35, 40]. In fact, AIF is believed to play a crucial role in the regulation of caspase-independent cell death [41]. In healthy cells, AIF is retained in the mitochondria, where it is believed to perform an oxidoreductase function; however, when AIF is released from the mitochondria in response to apoptotic stimuli, it becomes an active executioner of caspase-independent cell apoptosis [42]. Taken together, it becomes apparent that many cell types have recruited alternative caspase-independent mechanisms that come into play to ensure the completion of apoptosis which would be considered as advantage strategy in some pathologies such as cancer. Here, we demonstrated that the intrinsic apoptotic pathway triggered by IMP3 siRNA in CRC cells drives the nuclear translocation of AIF and the presence of AIF siRNA abrogates this programmed cell death activity. Apoptosis represents a key anti-cancer therapeutic effector mechanism. During apoptosis, mitochondrial outer membrane permeabilization typically kills cells, even in the absence of caspase activity. However, cancer cells are able to eradicate mitochondrial caspase-independent apoptosis machinery by up-regulating Bcl-2 anti-apoptotic proteins [30]. The identification of a regulator such as IMP3 could explain the molecular mechanism that allows the up-regulation of Bcl-2 antiapoptotic protein expression in colon cancer. Moreover, our results are consistent with the observation that the down-regulation of Bcl-2 and Bcl-xL suppresses the proliferation, migration and invasion of human CRC cell lines [30]. Caspase-independent cell death, triggered by the down-regulation of anti-apoptotic proteins such as Bcl-2, might be a potential candidate for such anti-cancer alternative therapeutic strategies. Recently, caspase-independent cell death has gained attention as a promising replacement for apoptosis in cancer treatment when Giampazolias et al. demonstrated that caspase-independent cell death triggers a sustained pro-inflammatory cytokine storm via NF-kB, leading to enhanced anti-tumoral activity compared to apoptosis [43, 44]. All this data, together with the demonstration that IMP3 knockdown sensitize CRC cells to anticancer drugs, open the way for CRC to be examined as a potential clinical target for anti-IMP3-based therapy. Moreover, one of the new potential chemotherapeutic approaches occurs through pro-apoptotic therapy [45, 46], therefore, the discovery of new player involved in alternative forms of programmed cell death, could open up novel and exciting perspectives to kill resistant cancer cells.

## Methods

### Patients and human samples

Samples of human CRC were derived from 32 patients (median age 65 *y*, range 44–82 *y*; 6 patients stage IV, 15 patients stage III, 8 patients stage II and 3 patients stage I) who had undergone colonic resection for sporadic CRC (T), whereas healthy (normal) mucosa samples included colonic mucosal biopsy from 18 patients (median age 57 *y*, range 38–77 *y*) with irritable bowel syndrome (CTR), from Tor Vergata University Hospital (Rome, Italy). In this cohort IMP3 expression was evaluated by Western blotting, while in paired tissue samples derived from the tumoral area (T) and the macroscopically unaffected, adjacent colonic mucosa (NT) of other 19 patients who underwent colon resection for sporadic CRC at the Tor Vergata University Hospital (Rome, Italy), by Western blotting and immunohistochemistry. Patients with sporadic CRC received neither radiotherapy nor chemotherapy before surgery. The study protocol was approved by the Tor Vergata University Hospital Review Board (protocol number 129/17).

### Cell culture

All reagents were purchased from Sigma-Aldrich unless otherwise specified. Human CRC cell lines HCT-116, HT-29 and DLD-1 were obtained from the American Type Culture Collection (ATCC, Manassas, VA) and cultured in McCoy’s 5 A (HCT-116, HT-29) and RPMI-1640 (DLD-1) medium. The medium was supplemented with 10% fetal bovine serum (FBS), 1% penicillin/streptomycin (all from Lonza, Verviers, Belgium). Human colon epithelial cell line (HCEC-1ct) was obtained from EVERCYTE GmbH (Vienna, Austria) and cultured in ColoUp medium (EVERCYTE GmbH) in low oxygen condition (2%). Cells were maintained in a 37 °C, 5% CO_2_, fully humidified incubator. Phosphorothioate single-stranded oligonucleotide of the human IMP3 complementary DNA sequence was synthesized in the antisense orientation (5’-TCGCTGAGGTTTCCGATATACA-3’) (Integrated DNA Technologies, Coralville, Iowa).

To assess effective IMP3 downregulation in vitro, cells were transfected for 24 h with 2 different commercial IMP3 siRNAs (with final concentration from 5 nmol/L to 25 nmol/L) and CTR siRNA (final concentration: 25 nmol/L) (all from Santa Cruz Biotechnology and Thermo Fisher Scientific) in presence or absence of 5-fluoruracil or Oxaliplatin (both at 100 nM final concentration) using Opti-MEM medium and lipofectamine 3000 reagent (both from Thermo Fisher Scientific), according to the manufacturer’s instructions. siRNA-mediated silencing of AIF was also performed with a commercial AIF-specific siRNA (Santa Cruz Biotechnology, final concentration: 25 nmol/L) for 24 h. As a nonspecific control, a scrambled siRNA was used (Santa Cruz Biotechnology). To assess the over-expression of Bcl-2 and Bcl-xL proteins in CRC cell lines we used the *Bcl-xL* and *Bcl-2* CRISPR Activation Plasmids according to the manufacturer’s instructions (Santa Cruz Biotechnology).

### Protein extraction and Western Blotting

Total proteins were extracted in lysis buffer as indicated before [47]. Membranes were incubated with antibodies against anti-human IMP3, phosphorylated (p)-RIP3, Gasdermin D (GSDMD), Glutathione peroxidase 4 (GPX-4) and cleaved caspase 3 (final dilution 1:1000, all from Abcam, Cambridge, UK), B-cell lymphoma (Bcl)-2 and Bcl-xL (1:500 final dilution, all from Santa Cruz Biotechnology, Santa Cruz, CA, USA). After the analysis, each blot was stripped and incubated with monoclonal β-actin antibody (1: 5000 final dilution) to ascertain equivalent loading of the lanes. Nuclear protein lysates, extracted as indicated before [48], were isolated from HCT-116-silenced cells and incubated with Apoptosis-inducing factor (AIF) and Octamer transcription factor 1 (OCT1) (1:500 final dilution, all from Santa Cruz Biotechnology) according to the manufacturer’s instructions. After primary antibody incubation all the membranes were incubated for 1 h with a secondary antibody conjugated to HRP (Dako, Milan, Italy).

Computer-assisted scanning densitometry was used to analyse the intensity of the immunoreactive bands for all Western blot panels.

### Immunohistochemistry

Immunohistochemistry was performed on cryosections of tumoral and macroscopically unaffected, adjacent colonic mucosa areas from CRC patients previously stained with haematoxylin and eosin. Immunohistochemical staining was performed using a monoclonal antibody directed against human IMP3 (final dilution 1:1000, Abcam) incubated at room temperature for 1 h and positive cells were visualized using MACH4 Universal HRP- Polymer kit with 3,3’-diaminobenzidine (DAB) (Biocare Medical, Pacheco, CA). Cryosections prepared from HCT-116-derived xenografts were stained with anti-IMP3 antibody (final diluition: 1:1000, Abcam). Isotype control sections were prepared under identical immunohistochemical conditions, replacing the primary antibody with a purified control isotype (IgG).

### TUNEL assay

To detect apoptotic cells in HCT-116-derived xenograft cryosections, the terminal deoxynucleotidyl transferase dUTP nick end labelling (TUNEL) assay was performed using the in situ cell death detection kit (Roche Applied Science, Penzberg, Germany) according to the manufacturer’s instructions. Apoptotic cells were analyzed by LEICA DMI4000 B microscope using LEICA application suite software (V4.6.2) and the percentage of tumour area covered by apoptotic cells was evaluated using ImageJ (NIH) software.

### Flow cytometry analysis

Cells were untreated or transfected with either control siRNA (final concentration 25 nmol/L) or IMP3 siRNA (final concentration 5 nmol/L to 25 nmol/L) and were incubated with Z-VAD-FMK (final concentration: 20 umol; R&D Systems, Minneapolis, MN) alone or in combination with deferoxamine (final concentration: 30umol) or disulfiram (final concentration: 10umol), or necrostatin-1 (final concentration: 30 umol). After 48 h, cells were collected, washed twice in Annexin V (AnnV) buffer, stained with FITC-AnnV (final dilution: 1:100; Immunotools, Friesoyte, Germany) according to the manufacturer’s instructions and incubated with 5 mg/mL of propidium iodide (PI) for 30 min at 4 °C. Cell death was quantified by flow cytometry; viable cells were considered as AnnV–/PI– cells. To assess mitochondrial membrane potential, tetraethylbenzimi-dazolylcarbocyanine iodide (JC-1) dye was used according to the manufacturer’s instructions (Thermo Fisher Scientific). We examined the activated form of Bax using a conformation-specific antibody that detects the active form of Bax (clone 6A7, final dilution1:100, Santa Cruz) as indicated before [49]. Analysis was performed using the Kaluza software (Beckman Coulter Life Sciences, Pasadena, CA).

### RNA extraction, RT-qPCR

RNA was extracted using PureLink mRNA mini kit (Thermo Fisher Scientific), according to the manufacturer’s instructions. A constant amount of RNA (1 μg/sample) was retrotranscribed into complementary DNA (cDNA). Reverse transcription was performed with Oligo (dT) primers and with M-MLV-reverse transcriptase (Thermo Fisher Scientific). Real-time PCR was performed using IQ SYBR Green Supermix (Bio-Rad Laboratories, Milan, Italy). RNA expression was calculated relative to the housekeeping *β-actin* gene (forward 5′- AAGATGACCCAGATCATGTTTGAGACC -3′, reverse 5′-AGCCAGTCCAGACGCAGGAT -3′) on the base of the Delta-DeltaCT algorithm. The sequences of *Bcl-2* and *Bcl-xL* primers used were as follows: forward 5′- AGGCTGGGATGCCTTTGTGGAA -3′, reverse 5′- CAAGCTCCCACCAGGGCCAAA -3′ and forward 5′- GGAGAACGGCGGCTGGGATA -3′, reverse 5′- GGCCACAGTCATGCCCGTCA-3′, respectively.

### RNA Immunoprecipitation

The RNA immunoprecipitation was assessed on HCT-116 and DLD-1 cells using the Magna RIP^TM^ RNA-binding protein immunoprecipitation kit, according to the manufacturer’s instructions (Merck KGaA, Darmstadt, Germany). Beads were incubated with a specific anti-IMP3 antibody (Signalway Antibody, Greenbelt, Maryland, USA) or anti-rabbit IgG. The co-immunoprecipitated RNA was extracted by TRIzol and analysed by RT-qPCR.

### In vivo formation of HCT-116-derived tumours

HCT-116 (5 × 10^5^) cells were resuspended in 500 ml BD Matrigel basement membrane matrix high concentration (BD Biosciences, Milan, Italy) and injected subcutaneously into the flank of 8-week-old female *Rag1*^-/-^ mice, whose fur was previously shaved and depilated. After 1 week, by calliper measurements mice with similar tumour volumes were divided into two groups receiving daily intraperitoneal injection of either control sense (S) or IMP3 anti-sense oligonucleotide (AS, both at concentration of 100 μg/mouse resuspended in 300 ml of PBS). Mice were sacrificed at day 21. Tumours were excised and photographed, their volume was calculated according to the following formula: 1/2x(short diameter)x(long diameter)x(height) and entirely included in cryostat mounting medium (Thermo Fisher Scientific) for following histochemistry experiments. To assess the successful uptake of the IMP3 AS in the growing tumours, in preliminary experiments mice were intraperitoneally injected with fluorescein isothiocyanate (FITC)-conjugated IMP3 AS (100 μg/mouse) or PBS as control. Twenty-four hours later, mice were killed, tumours were excised and included in cryostat mounting medium to obtain frozen sections. FITC-conjugated IMP3 AS distribution was assessed by immunofluorescence. All experiments using animals were performed according to Italian and European legislation on animal experimentation (protocol number: 494/2017-PR).

### Immunofluorescence

Immunofluorescence was performed on frozen sections of HCT-116-derived tumours of FITC-coniugated IMP3 AS treated-mice. All tumours were completely embedded in a cryostat mounting medium (Thermo Fisher Scientific), snap-frozen and stored at −80 °C; 6 μm-thickened sections were mounted onto superfrost plus glass slides. After three washes in PBS, coverslips were mounted on glass slides using ProLong Gold antifade reagent with 4′,6-diamidino-2-phenylindole (DAPI) (Thermo Fisher Scientific) to counterstain the DNA. Images were acquired on a Leica DMI 4000 B fluorescence microscope (Leica, Wetzlar, Germany).

### Statistical analysis

Values are expressed as mean ± standard deviation (SD) or ± standard error of the mean (SEM). Statistical analysis of the data was performed using the Student t test and Mann-Whitney test. GraphPad Prism 6 (GraphPad Software, La Jolla, CA) was used for statistical and graphical data evaluations. *P* values < 0.05 were considered statistically significant.

## Supplementary information

Supplemental Figures

WB uncropped

Checklist

## Data Availability

All raw data that support the findings of this study are available from the corresponding author upon reasonable request. The data available in public repository that support the findings of this study are available in Tumour Immune Estimation Resource (TIMER) http://timer.cistrome.org/, the Gene Expression Profiling Interactive Analysis (GEPIA) http://gepia.cancer-pku.cn/index.html, (UALCAN) http://ualcan.path.uab.edu/, (Human Protein Atlas database) https://www.proteinatlas.org/ and Post-transcriptional Regulation (POSTAR3) http://111.198.139.65/index.html.
