# Supplementary material for: A novel tumour enhancer function of Insulin-like growth factor II mRNA-binding protein 3 in colorectal cancer
Source: Cell Death Dis. 2023 Apr 6;14(4):243. doi: 10.1038/s41419-023-05772-6 (PMC10079693; doi:10.1038/s41419-023-05772-6)

Supplementary figure 1: Expression of IMP3 in CRC. (A) IMP3 mRNA expression in different types of cancer was investigated with the TIMER database. Circle normal colon tissue vs CRC tissue. (B and C) Increased mRNA expression of IMP3 in CRC tissue compared to normal tissues by GEPIA (B) and ULCAN (C) database.

Supplementary figure 2. (A) Upper panel, representative Western blot showing IMP3 expression in HCT-116 cells untreated (Unst) or transfected with control siRNA oligonucleotide (CTR siRNA) or specific IMP3 siRNA (IMP3 siRNA2, final concentration 25 nM). Lower left panel, representative dot plot of Annexin V (AnnV) and propidium iodide (PI)-positive HCT-116 cells treated as indicated above for 48 hours. Lower right panel, quantification of the percentage of AnnV and/or PI-positive HCT-116 cells (mean ± SEM; AnnV+PI+ unst and CTR sense-treated cells versus IMP3 AS transfected cells, $^*p \leq 0.06$, $n = 3$). (B) Representative dot plot and percentage of Annexin V (AnnV) and propidium iodide (PI)-positive DLD1 (upper panels) ad HT-29 (lower panels) cells untreated (Unst) or transfected with control or IMP3 siRNA (both final concentration 25nM) for 48 hours. (mean ± SEM; AnnV+PI+ unst and CTR sense-treated cells versus IMP3 AS transfected cells, $^*p \leq 0.06$, $n = 3$). (C) Left panel, representative dot plot of Annexin V (AnnV) and propidium iodide (PI)-positive HCEC-1ct cells untreated (Unst) or transfected with control or IMP3 siRNA (both final concentration 25nM) 48 hours. Lower right panel, quantification of the percentage of AnnV and/or PI-positive HCEC-1ct cells (mean ± SEM; AnnV+PI+ unst and CTR sense-treated cells versus IMP3 AS transfected cells, $^*p \leq 0.06$, $n = 3$). (D) Representative Western blotting of procaspase and activated form of caspase 8 and 9 in HCT-116 cells left untreated (Unst) or transfected with control or IMP3 siRNA for 36 hours. β-actin was used as loading control.

Supplementary figure 3. Representative dot plot (upper panels) and percentage quantification (lower panel) of Annexin V (AnnV) and propidium iodide (PI)-positive HCT-116 cells were pre-incubated with pan-caspase inhibitor (Z-VAD) alone or with deferoxamine (Def, final concentration: 30umol) or disulfiram (Dis, final concentration: 10umol), or necrostatin-1 (Nec, final concentration: 30umol) and were left untreated (Unst) or transfected with control or IMP3 siRNA for 48 hours. Staurosporin (Stauro), LPS, Fe^{3+} and TNF-α was used as positive control. n=2.

Supplementary figure 4. *Bcl-2* and *Bcl-xL* mRNA are part of the IMP3 complex. Left, representative Western blotting of IMP3 immunoprecipitation from DLD-1 cells, β-actin is used as a negative control. Input (1:10) of the total protein extracts, IMP3 immunoprecipitation (IP: IMP3), and mock immunoprecipitation (IP: IgG). Right, relative quantification of IMP3-mRNAs enrichment by PCR in IMP3 immunoprecipitation/total protein extracted from DLD-1 cells. *β-actin* mRNAs were used as control (mean ± SEM; IP: IMP3 versus IP: IgG *P ≤ 0.05; n=3).

Supplementary figure 5. (A) Upper panel, representative dot plot of Annexin V (AnnV) and propidium iodide (PI)-positive HCT-116 cells untreated (Unst) or transfected with control siRNA oligonucleotide (CTR siRNA) or specific Bcl-2 siRNA and/or specific Bcl-xL siRNA (final concentration 25 nM). Lower right panel, quantification of the percentage of AnnV and/or PI-positive HCT-116 cells (mean ± SEM; unst and CTR sense-treated cells versus BCl-2 or Bcl-xL siRNA , *p ≤ 0.04, n = 3). (B) Upper panel, representative dot plot of Annexin V (AnnV) and propidium iodide (PI)-positive HCT-116 cells untreated (Unst) or transfected with or specific Bcl-2 CRISPR Activation Plasmids (Bcl-2 P) and specific Bcl-xL CRISPR Activation Plasmids (Bcl-xL p, final concentration 2 ug/ml) alone or with specific IMP3 siRNA for 48 hours. Lower right panel, quantification of the percentage of AnnV and/or PI-positive

HCT-116 cells (mean ± SEM; BCl-2 p+ Bcl-xL è vs BCl-2 p+ Bcl-xL+IMP3 siRNA, **p ≤ 0.01, n = 3).

Supplementary figure 6. (A) Reppresentative flow cytometry measurement (upper panels) and percentage quantification (lower panel) of the active conformation of Bax in HCT116 cells unstimulated (Unst) or transfected with control or IMP3 siRNA for 30 hours (mean ± SEM; *p= 0.01; IMP3 siRNA versus CTR sense, n=2). (B) Representative Western blot showing AIF expression in HCT-116 cells untreated (Unst) or transfected with CTR or IMP3 siRNA in presence/absence of AIF siRNA for 48 hours.

Supplementary Figure 1

A

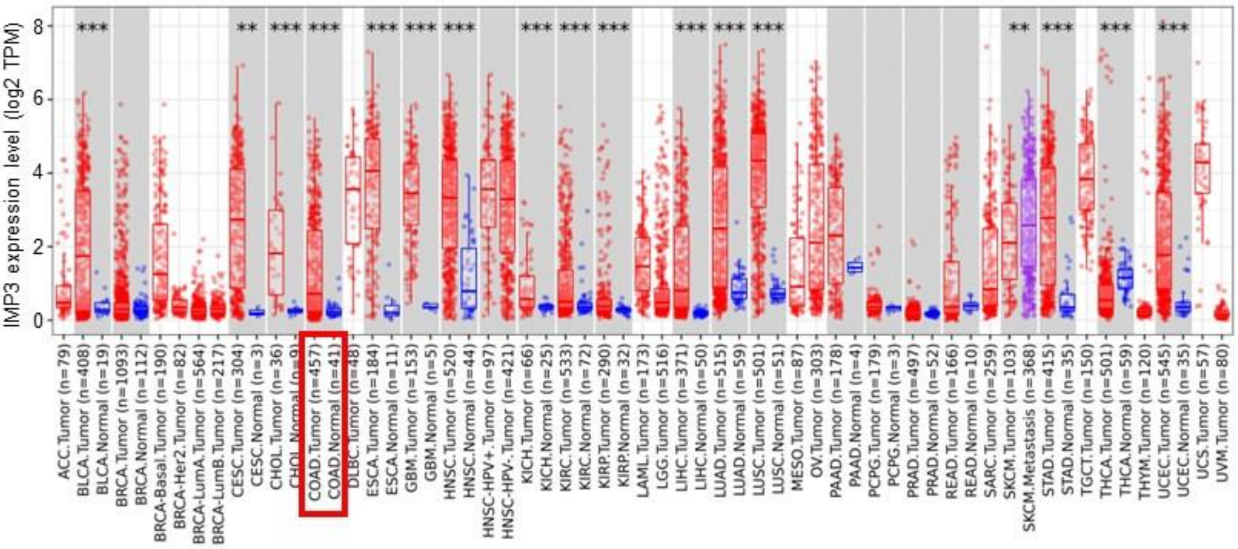

B

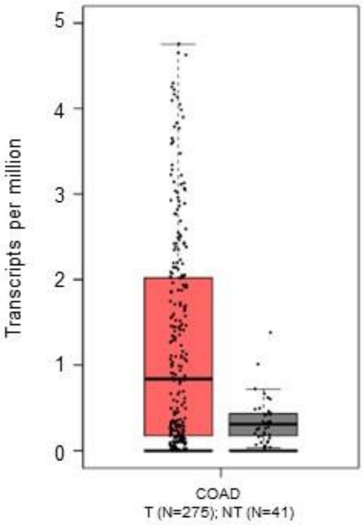

C

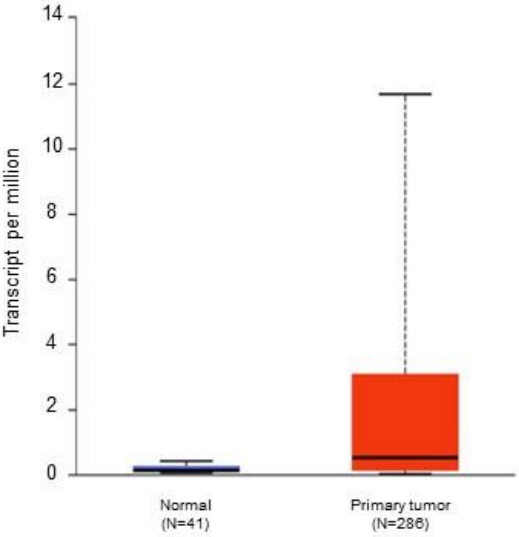

Supplementary Figure 2

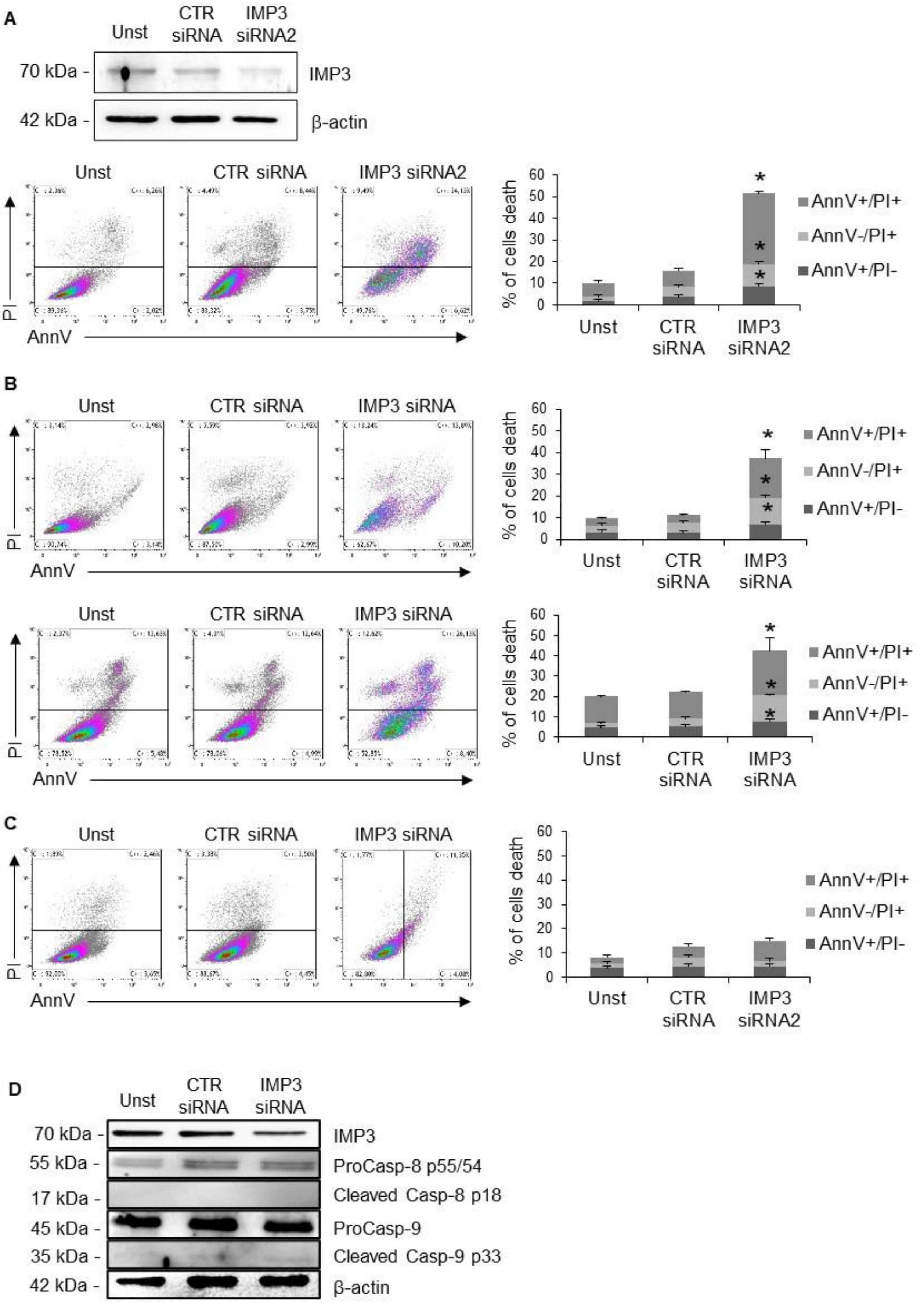

Supplementary Figure 3

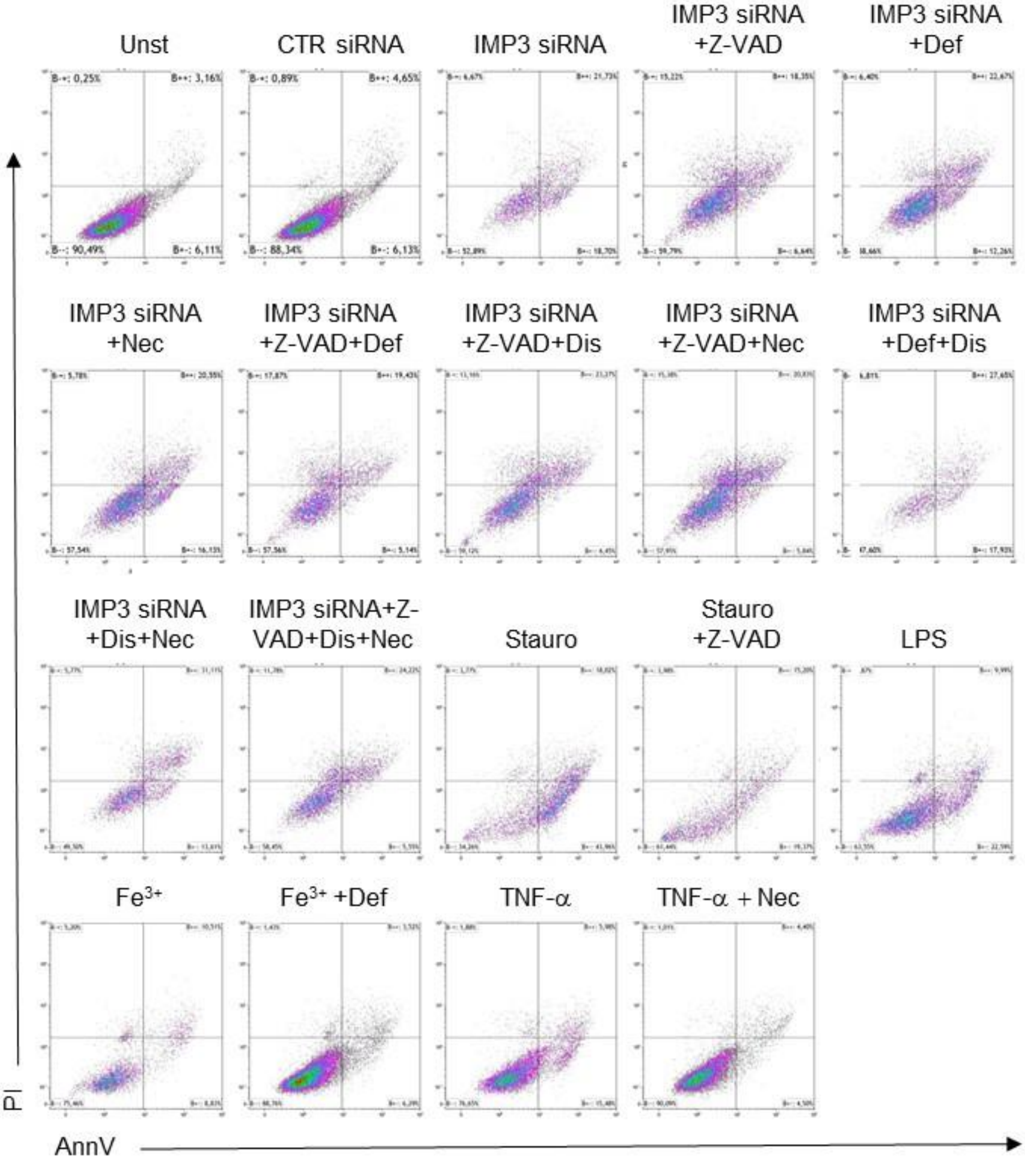

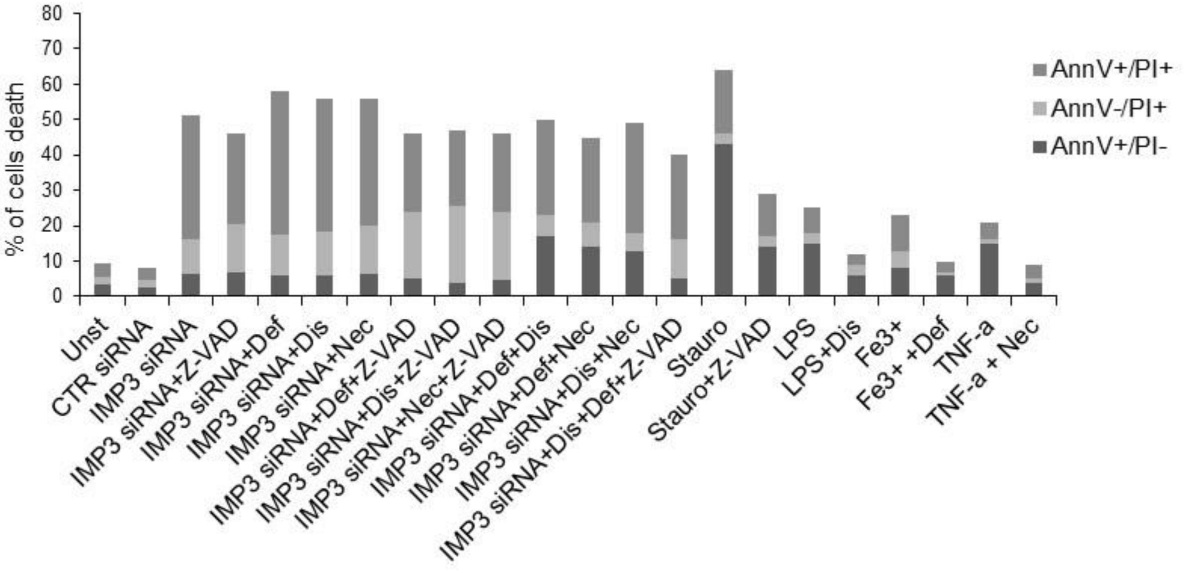

Supplementary Figure 4

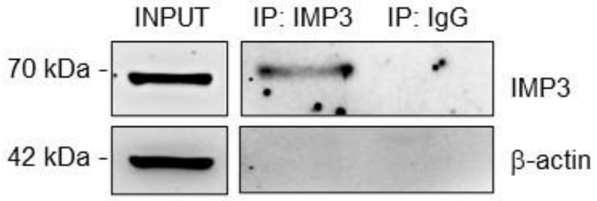

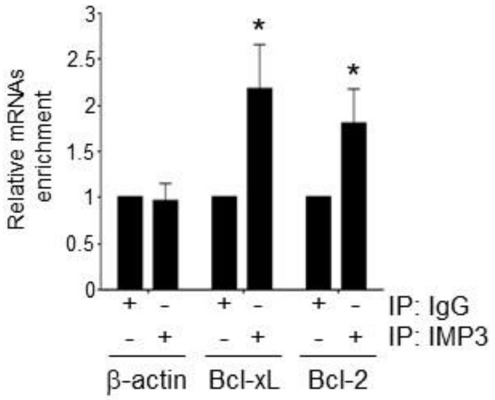

Supplementary Figure 5

A

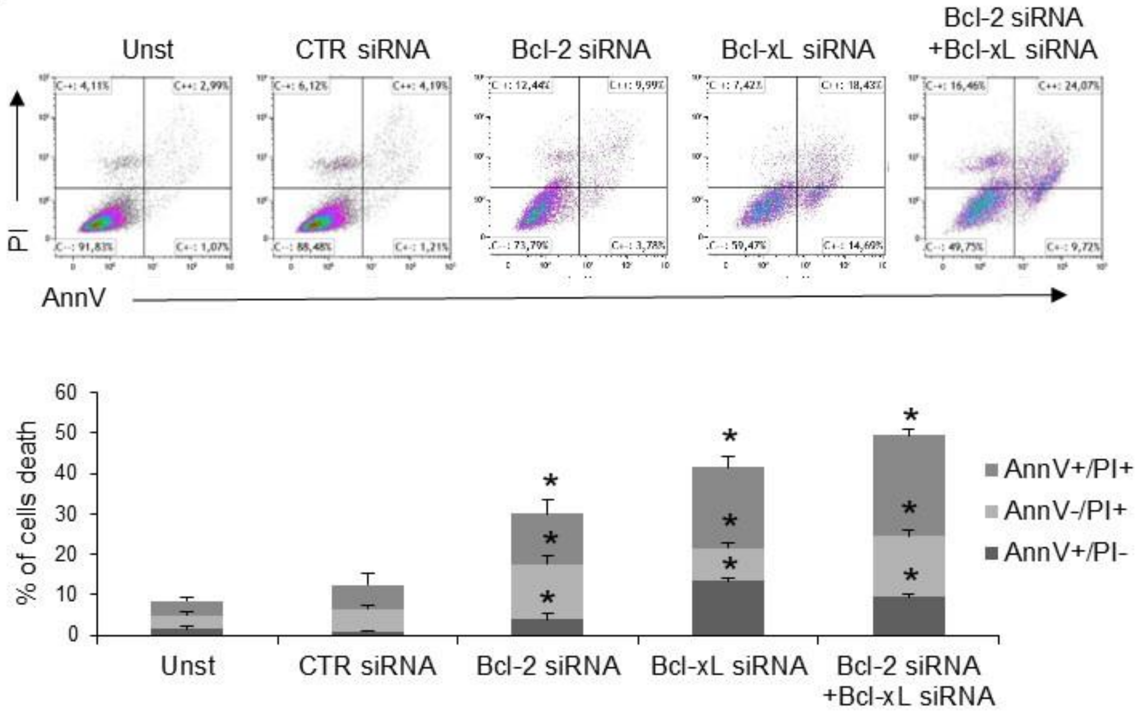

B

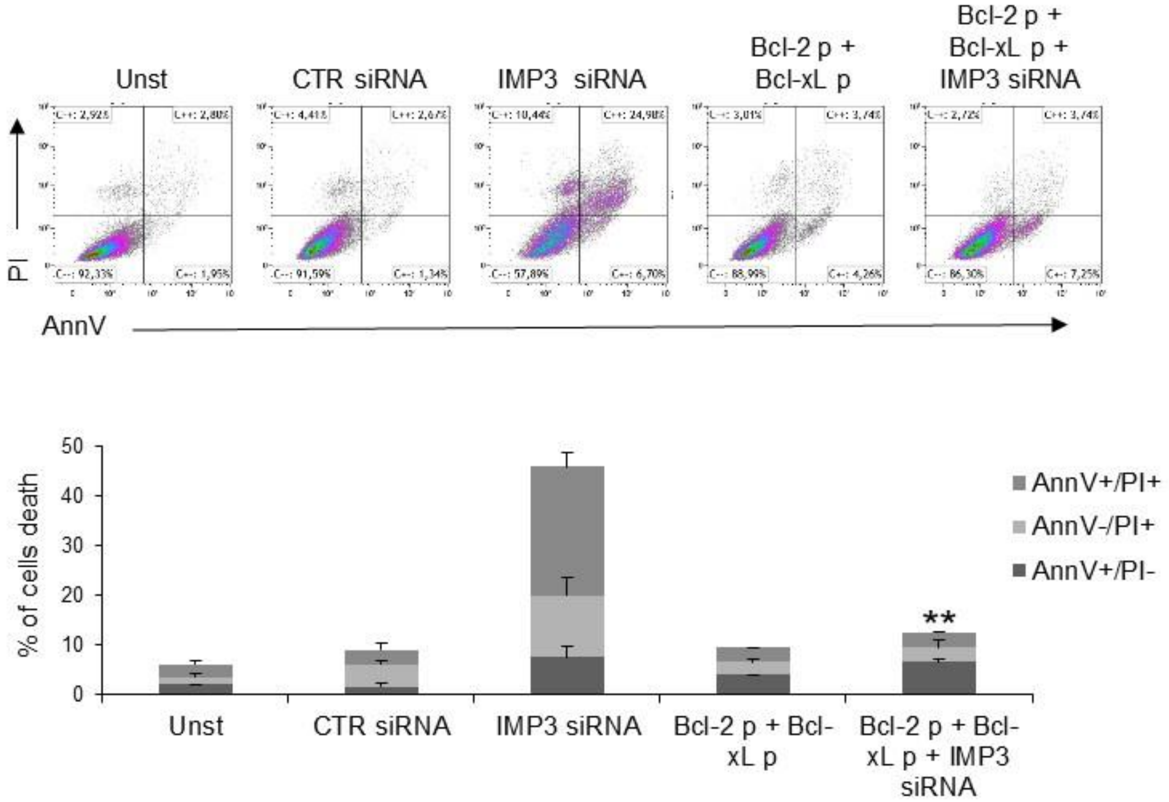

Supplementary Figure 6

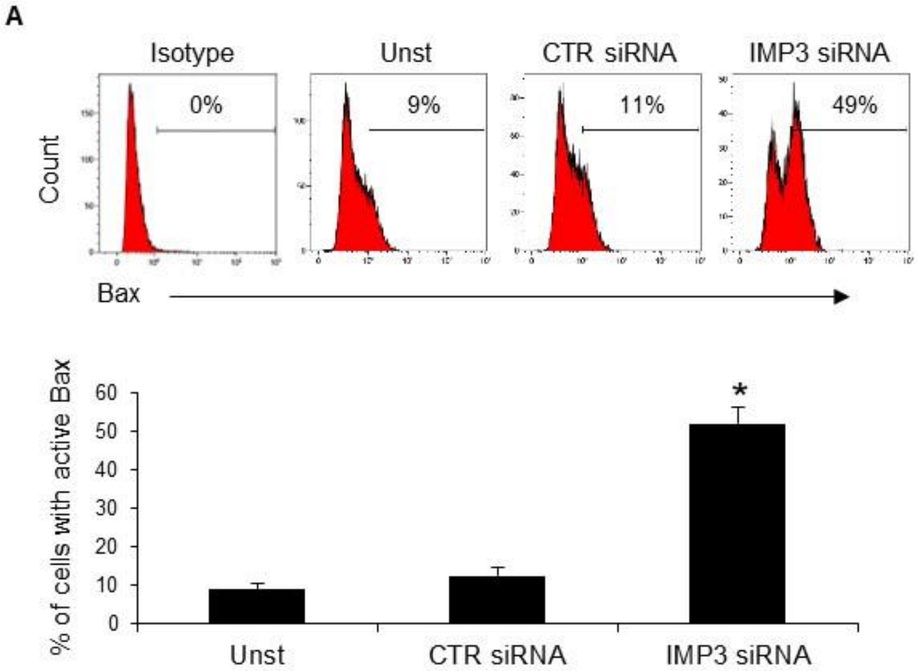

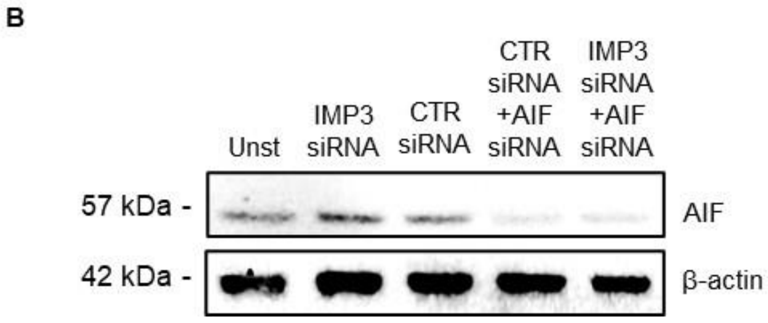

Supplement: Supplementary file 1 — Supplemental Figures [file 41419_2023_5772_MOESM1_ESM.pdf]
