# Supplementary material for: A novel tumour enhancer function of Insulin-like growth factor II mRNA-binding protein 3 in colorectal cancer
Source: Cell Death Dis. 2023 Apr 6;14(4):243. doi: 10.1038/s41419-023-05772-6 (PMC10079693; doi:10.1038/s41419-023-05772-6)
Supplement: Supplementary file 2 — WB uncropped [file 41419_2023_5772_MOESM2_ESM.pdf]

Figure 1A

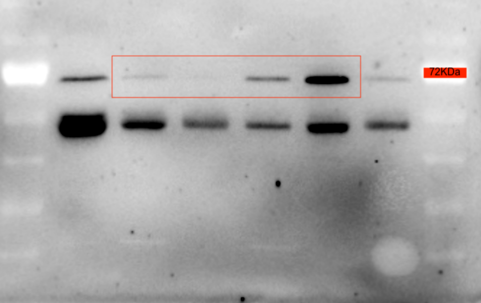

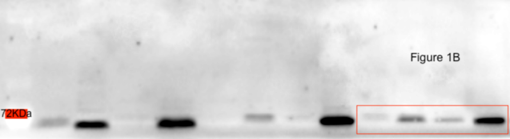


Figure 1B

Figure 2A

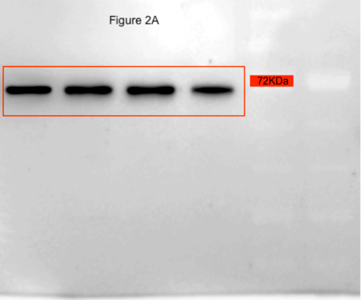

Figure 2B

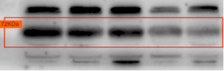

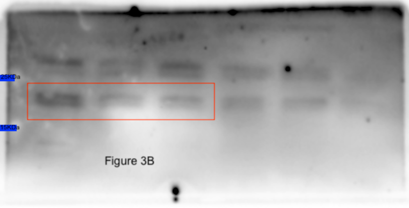


Figure 3B

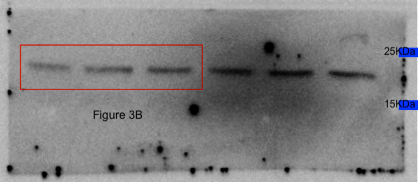
25KDa
Figure 3B
15KDa

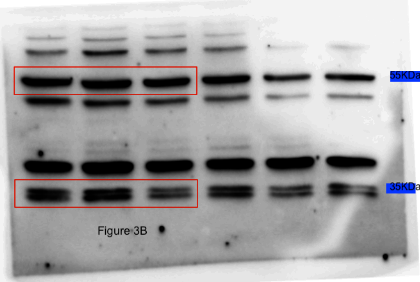


Figure 3B

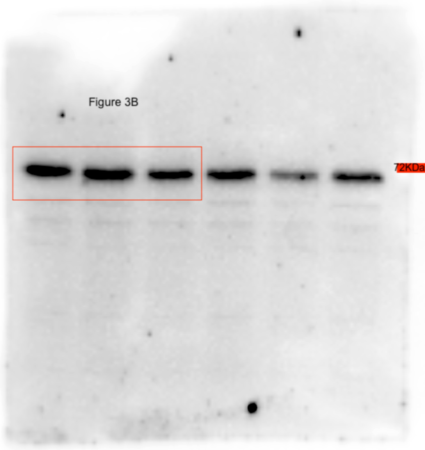

Figure 3B
72KDa

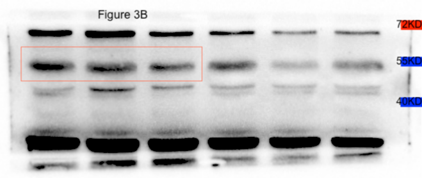

Figure 3B
72KD
55KD
40KD

Figure 4A

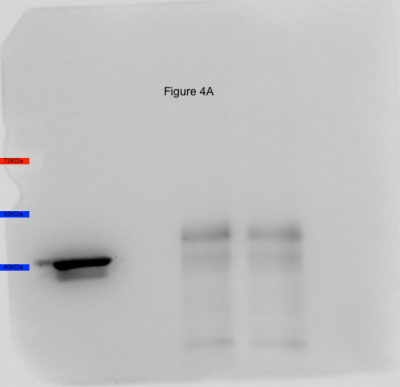

Figure 4A

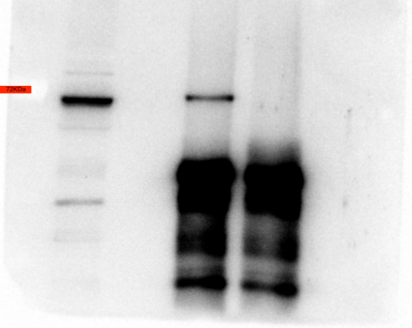

Figure 4C

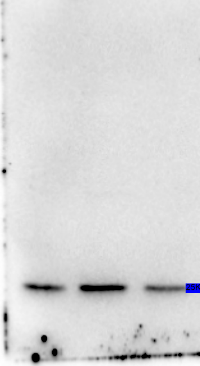

Figure 4C

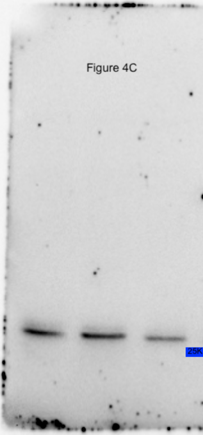

Figure 4C

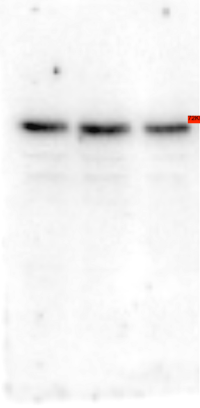

Figure 5B

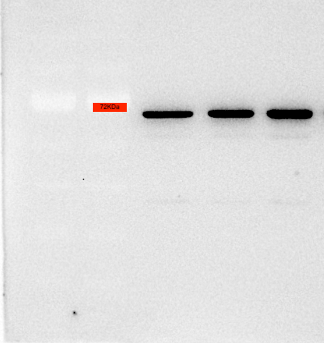

Figure 5B

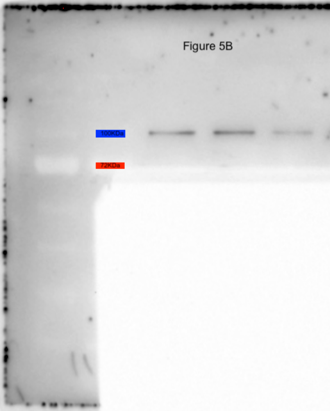

# Fig6c

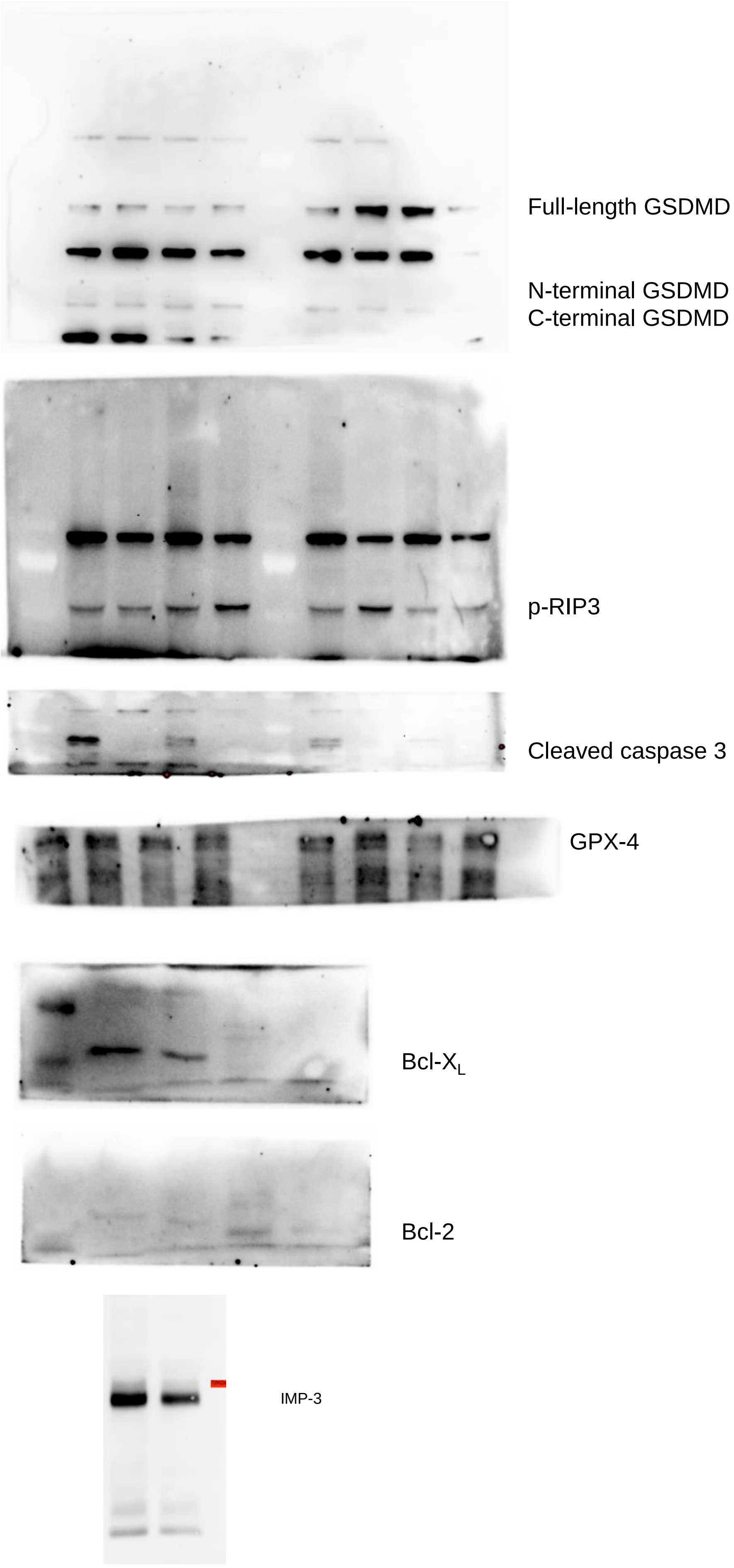

Full-length GSDMD
N-terminal GSDMD
C-terminal GSDMD
p-RIP3
Cleaved caspase 3
GPX-4
Bcl-$X_L$
Bcl-2
IMP-3

FigS3a

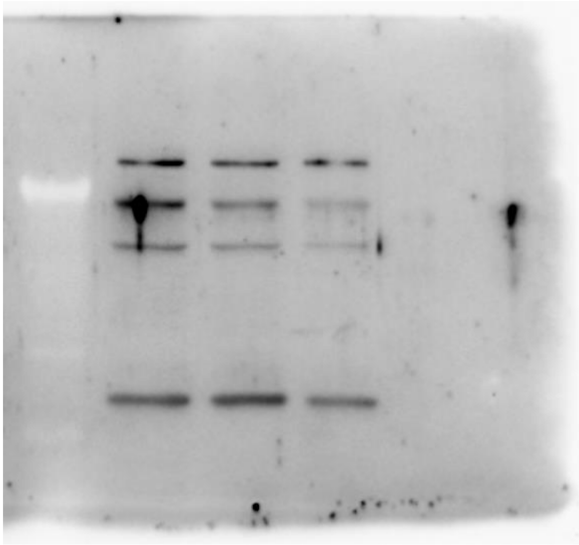

imp3

FigS6

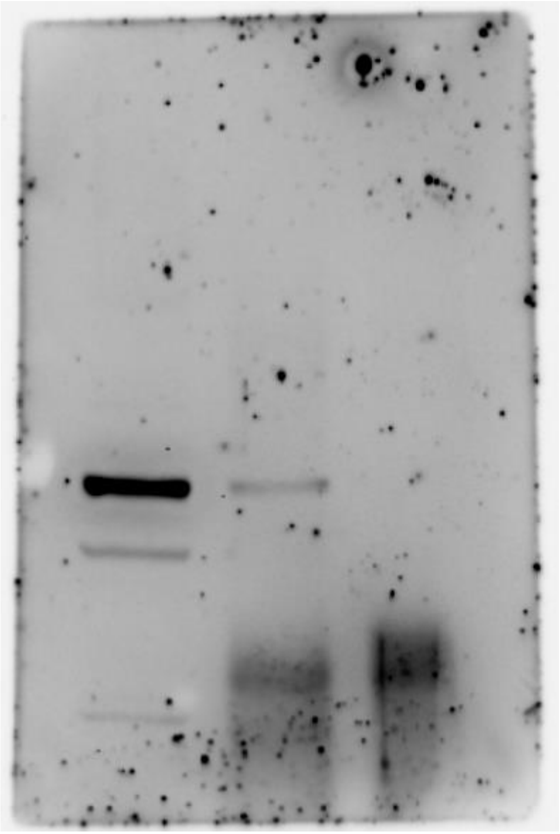

imp3

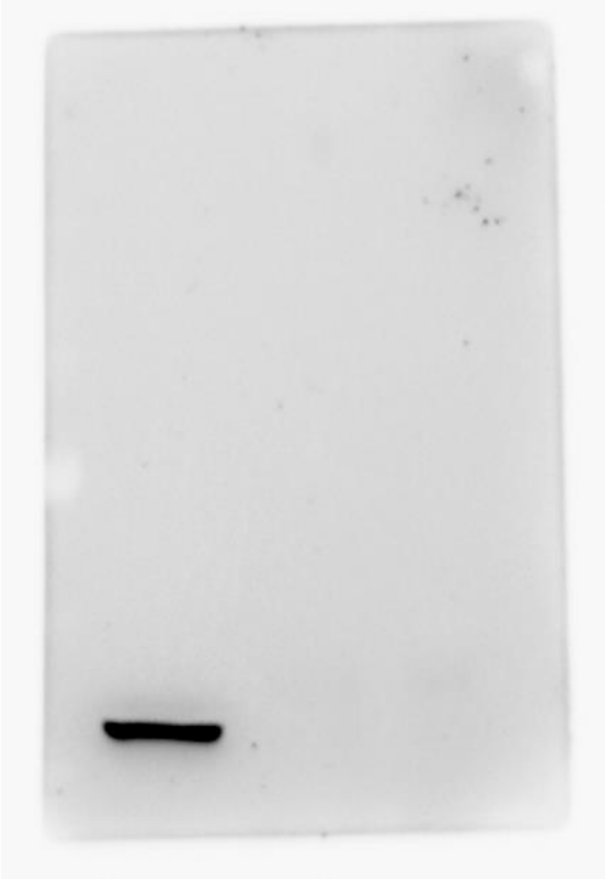

Actin

Figs8b

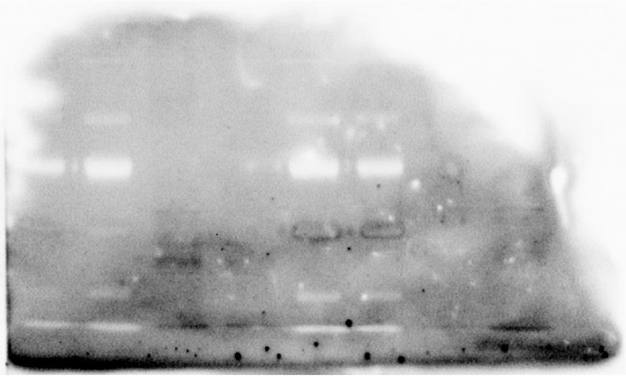

Cytochrome c

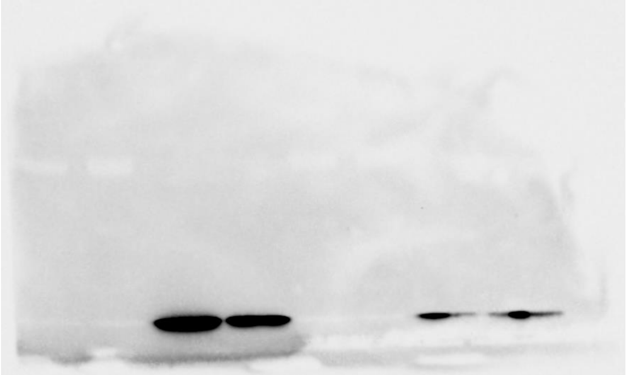

Cox IV

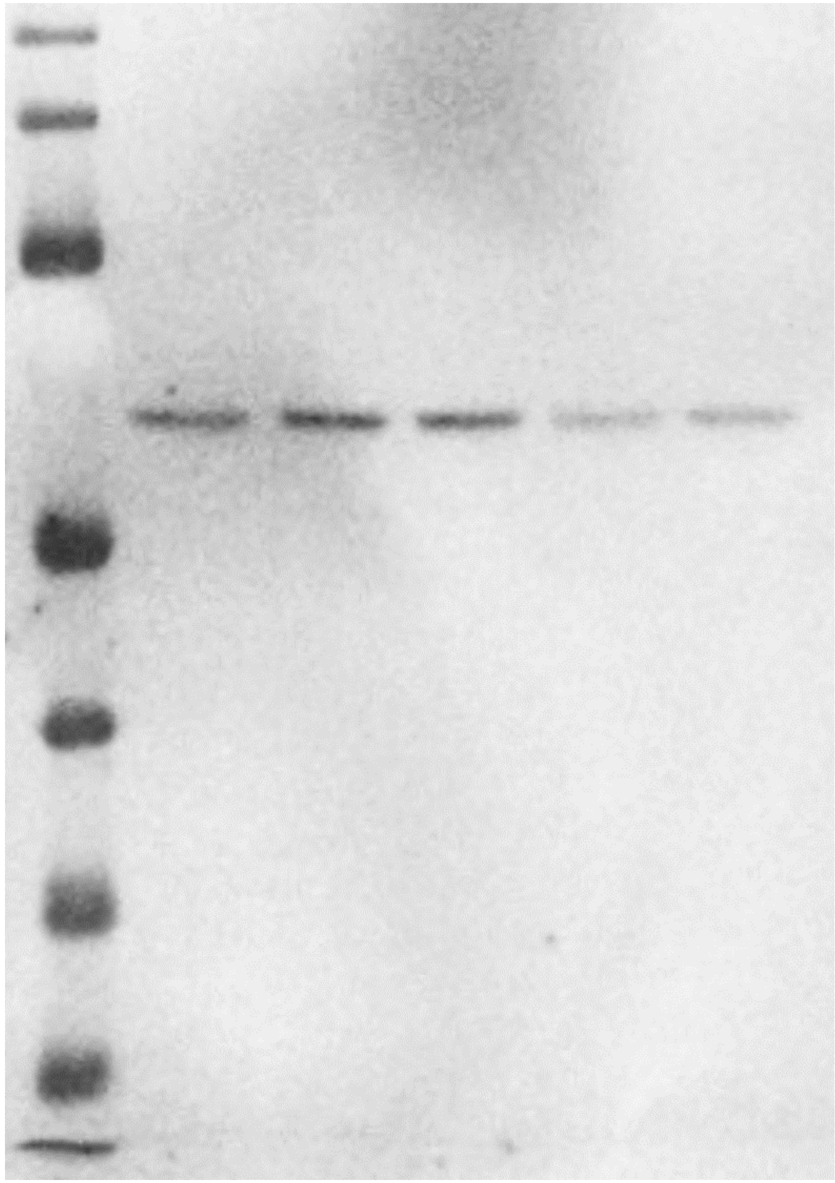

AIF

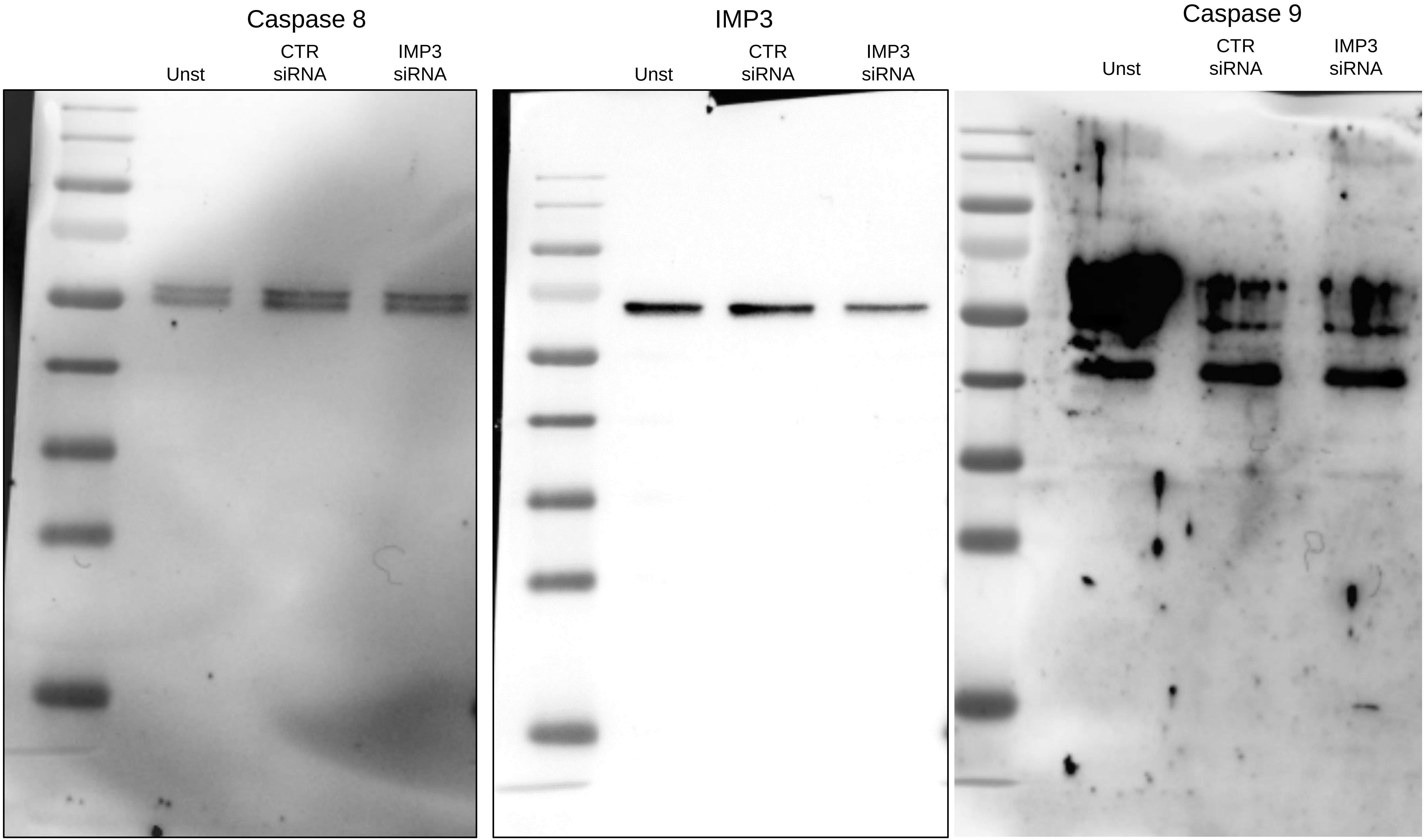
Caspase 8
Unst
CTR siRNA
IMP3 siRNA
IMP3
Unst
CTR siRNA
IMP3 siRNA
Caspase 9
Unst
CTR siRNA
IMP3 siRNA
